# CSE/H_2_S/SESN2 Signalling Mediates the Protective Effect of Exercise Against Immobilization‐Induced Muscle Atrophy in Mice

**DOI:** 10.1002/jcsm.70083

**Published:** 2025-10-01

**Authors:** Xiuru Li, Yating Huang, Xuege Yang, Sujuan Liu, Yanmei Niu, Li Fu

**Affiliations:** ^1^ Department of Rehabilitation, School of Medical Technology Tianjin Medical University Tianjin China; ^2^ Department of Histology and Developmental Biology, School of Basic Medical Science Tianjin Medical University Tianjin China; ^3^ Department of Physiology and Pathophysiology, School of Basic Medical Science Tianjin Medical University Tianjin China

**Keywords:** cystathionine‐γ‐lyase, exercise, hydrogen sulphide, muscle atrophy, oxidative stress, SESN2

## Abstract

**Background:**

Hydrogen sulphide (H_2_S), a gasotransmitter synthesized by cystathionine‐γ‐lyase (CSE), exhibits antioxidant properties and may mimic exercise‐induced muscle protection. However, its mechanistic role in muscle atrophy and exercise intervention remains unclear.

**Methods:**

Six‐month‐old male wild‐type (WT) and SESN2 knockout (SESN2^−/−^) C57BL/6J mice were subjected to a 2‐week hindlimb immobilization, followed by combined resistance and aerobic exercise or pharmacological intervention using the H_2_S donor NaHS (30 μmol/kg) or the CSE inhibitor DL‐propargylglycine (PAG, 50 mg/kg). In vitro, C_2_C_12_ myotubes were treated with H_2_O_2_ and NaHS to assess oxidative stress injury. Muscle mass, cross‐sectional area (CSA), collagen deposition and oxidative stress markers were evaluated via histology, Western blot and immunofluorescence.

**Results:**

Compared with the immobilization (IM) group, mice receiving a 2‐week combined exercise intervention (IM + EX) exhibited significantly increased gastrocnemius muscle mass/body weight (10.86 ± 0.62 vs. 8.56 ± 1.61, *p* < 0.01), enlarged muscle fibre CSA (1628 ± 265 μm^2^ vs. 905.5 ± 88.52 μm^2^, *p* < 0.01) and reduced collagen deposition as indicated by Sirius red staining (collagen‐positive area: 2.86% ± 1.12% vs. 7.06 ± 1.18%, *p* < 0.001). Pharmacological inhibition of CSE with PAG significantly attenuated these exercise‐induced improvements (muscle mass/body weight: 10.22 ± 0.59, CSA: 1139 ± 96.21 μm^2^, collagen area: 5.04 ± 0.66%, all *p* < 0.05 vs. IM + EX). Conversely, administration of the H_2_S donor NaHS mimicked the protective effects of exercise, increasing muscle mass/body weight (8.94 ± 0.51), CSA (1474 ± 176.1 μm^2^) and reducing collagen accumulation (collagen area: 3.04 ± 0.74%, all *p* < 0.05 vs. IM). In vitro, NaHS treatment (30 μM) significantly reversed H_2_O_2_‐induced reductions in myotube diameter (19.16 ± 0.91 μm vs. 15.61 ± 0.72 μm, *p* < 0.01) and improved fusion index (46.47 ± 1.51% vs. 35.28 ± 2.87%, *p* < 0.05). Western blot analysis showed that NaHS upregulated SESN2 and Nrf2 expression, as well as downstream antioxidant proteins HO‐1 and NQO1 (*p* < 0.05), whereas SESN2 knockdown blocked these effects and abolished NaHS‐mediated protection in myotubes. In SESN2^−/−^ mice, NaHS failed to increase muscle mass/body weight (7.24 ± 1.3 vs. WT + NaHS 10.12 ± 0.38, *p* < 0.001), CSA (699.2 ± 21.51 μm^2^ vs. WT + NaHS 1189 ± 93.27 μm^2^, *p* < 0.001) or antioxidant capacity, confirming the essential role of SESN2 in mediating H_2_S‐dependent muscle protection.

**Conclusions:**

H_2_S protects against disuse‐induced muscle atrophy by enhancing antioxidant defences via the SESN2/Nrf2 signalling pathway. These findings identify H_2_S as a potential exercise‐mimetic therapeutic strategy for preserving muscle mass and function.

## 1 Introduction

Skeletal muscle, which constitutes 40%–50% of total body mass, plays a crucial role in essential bodily functions, including respiration, physical activity and blood glucose regulation, highlighting the importance of maintaining healthy muscle [1]. Prolonged muscle immobilization (e.g., bed rest, loss of motor neurons or limb immobilization) can lead to muscle atrophy, commonly referred to as disuse muscle atrophy [2]. In addition to aggravating existing medical conditions, disuse muscle atrophy increases the risk of falls, weakness, joint instability and even mortality [3]. Exercise remains the only effective intervention for preventing or reversing many forms of muscle atrophy [4, 5]. However, the precise effects of early exercise intervention on disuse muscle atrophy and the underlying mechanisms remain poorly understood.

Hydrogen sulphide (H_2_S) is an endogenous gas, identified after nitric oxide (NO) and carbon monoxide (CO), and is synthesized in various tissues, including the heart, liver, kidneys, blood vessels, brain, gastrointestinal tract and skeletal muscle [6]. In mammals, H_2_S is primarily synthesized by cystathionine‐γ‐lyase (CSE), cystathionine‐β‐synthase (CBS) and 3‐mercaptopyruvate sulphur transferase (3‐MST), with CSE being the predominant enzyme responsible for H_2_S production in skeletal muscle [7, 8]. A study demonstrated that 2 weeks of hindlimb immobilization induced oxidative stress and muscle atrophy, whereas H_2_S donors mitigated these effects [9]. Similarly, in obesity and dexamethasone‐induced muscle atrophy models, skeletal muscle CSE/H_2_S expression was significantly downregulated and H_2_S donors alleviated muscle atrophy symptoms [10, 11, 12]. Notably, exercise has been shown to improve glucose metabolism, reduce hepatic lipid accumulation and decrease inflammatory responses by enhancing CSE/H_2_S pathways across multiple organs [13, 14]. These findings underscore the potential role of the CSE/H_2_S in maintaining skeletal muscle mass and function. However, research on CSE/H_2_S expression during disuse muscle atrophy, as well as its role in exercise‐mediated regulation, remains limited.

Sestrins (SESNs) are a family of stress‐inducible proteins in vertebrates, comprising SESN1, SESN2 and SESN3 [15]. Our previous studies identified SESNs as mediators of exercise benefits, including enhanced physical endurance, improved glucose uptake and white adipose tissue browning [16, 17, 18]. Additionally, SESN2 mediated the protective effects of exercise against dexamethasone‐ and immobilization‐induced muscle atrophy [19, 20], suggesting that SESN2 could be a promising therapeutic target for muscle atrophy. In this study, we investigated the impact of early exercise intervention (1 week after immobilization) on disuse muscle atrophy and its effects on skeletal muscle CSE/H_2_S expression. Furthermore, we assessed the role of the systemic CSE/H_2_S pathway in the exercise‐mediated modulation of disuse muscle atrophy using CSE inhibitors and H_2_S donors. In the cell and animal model, we also compared the effects of H_2_S donor intervention with SESN2 knockdown on muscle atrophy.

## 2 Materials and Methods

### 2.1 Animals

Six‐month‐old male wild‐type (WT) and SESN2 knockout (SESN2^−/−^) C57BL/6J mice were used for all in vivo experiments. WT mice were obtained from SPF Biotechnology Co. Ltd. (Beijing, China). SESN2^−/−^ mice were generated by GemPharmatech Co. Ltd. (Nanjing, China) using the CRISPR/Cas9 system. Mice were housed under a 12‐h light/dark cycle at 22°C ± 2°C and 40%–60% humidity, with free access to food and water. All animal protocols were approved by the Tianjin Medical University Animal Care and Use Committee (Approval Number: SYXK‐2019‐0004).

### 2.2 Muscle Atrophy Model and Exercise Protocol

To investigate the effects of exercise on disuse muscle atrophy, mice were randomly assigned to three groups: sedentary control (CON), immobilization (IM) and immobilization plus exercise (IM + EX). Immobilization was performed as previously described [20, 21], with both hindlimbs positioned in knee extension and plantar flexion and wrapped in a 12‐ to 15‐mm‐wide cast for 2 weeks. The exercise intervention followed our previous study [20]. The exercise protocol is detailed in Data S1.

### 2.3 Others

All details of the methods are shown in Data S1.

## 3 Results

### 3.1 Hindlimbs Immobilization Resulted in Weight Loss and Reduced Exercise Capacity

The study protocol is illustrated in Figure S1A, with cast immobilization depicted in Figure S1B and aerobic/resistance exercise training shown in Figure S1C–D. To induce atrophy, the hindlimbs of mice were immobilized in a knee extension and ankle plantarflexion position for 2 weeks. In this position, the posterior calf muscles, particularly the gastrocnemius and soleus muscles, were predominantly shortened. As expected, immobilization resulted in weight loss (Figure S2A). Notably, a reduction in food intake was only observed during the first 2 days of immobilization (Figure S2B), with no significant difference in total food intake over the immobilization period (Figure S2C). This indicates that the weight loss was not attributable to malnutrition. We also assessed the impact of immobilization on locomotor ability. The maximal voluntary carrying capacity (MVCC) test revealed a reduction in muscle strength, irrespective of whether it was normalized to body weight (Figure S2D). Immobilization also decreased performance in the inverted screen tests and accelerating rotarod tests (Figure S2E,F), suggesting impairments in motor coordination and balance. Our previous study demonstrated a reduction in the index of muscle mass (IMM) and the cross‐sectional area (CSA) of gastrocnemius muscle fibres following 2 weeks of immobilization [20]. These findings collectively suggest that 2 weeks of immobilization results in reduced calf mobility and atrophy of the posterior muscle groups.

### 3.2 The Impact of Combined Exercise on Muscle Function and Quality Following Immobilization‐Induced Atrophy

**FIGURE 1:**
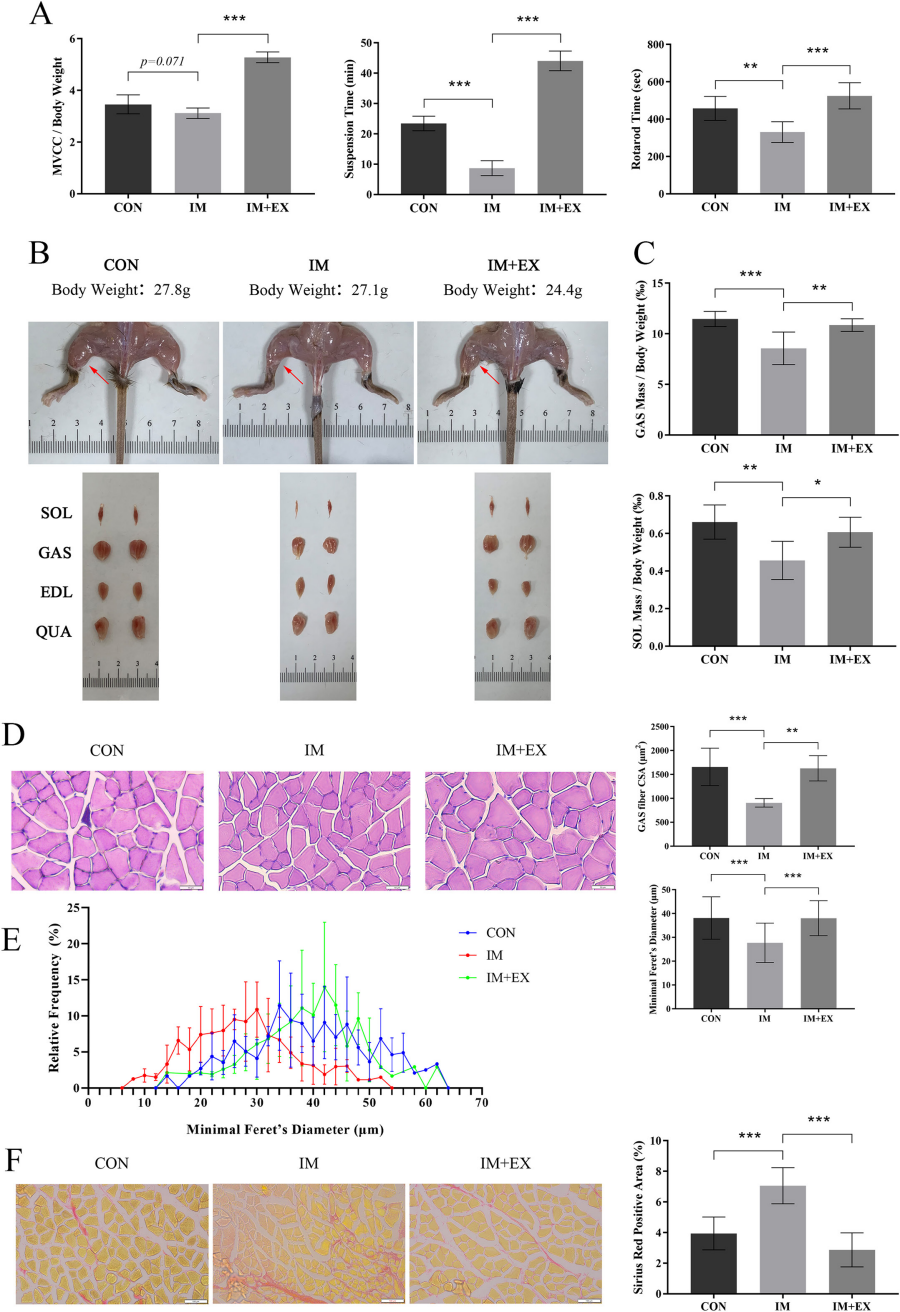
Effects of combined exercise on muscle function and mass in immobilization‐induced muscle atrophy mice. (A) Muscle function measurement. (B) Representative images of hindlimb morphology and muscles. (C) GAS and SOL muscle mass adjusted for body weight. (D) Haematoxylin and eosin (H&E) staining of the GAS muscle. Scale bar: 50 μm. Cross‐sectional area (CSA) and minimum Feret's diameter of GAS myofibers were analysed using Image J. (E) Distribution of GAS myofiber diameters. (F) Sirius red staining of the GAS muscle. Scale bar: 100 μm. The proportion of Sirius red‐positive areas in the GAS muscle was analysed using Image J. One‐way ANOVA was used for A, C, D and F (*n* = 6). Values are means ± SEM *p < 0.05; **p < 0.01; ***p < 0.001. EDL: extensor digitorum longus; GAS: gastrocnemius; QUA: quadriceps; SOL: soleus.

To assess the effects of exercise on immobilization‐induced muscle atrophy, mice in the IM group were allowed to recover naturally for 3 weeks following 2 weeks of cast immobilization. In contrast, mice in the IM + EX group underwent natural recovery for 1 week followed by 2 weeks of combined exercise training (Figure S1A). We examined the effects of combined exercise on muscle function and mass. Although muscle strength, coordination and balance in the IM group partially rebounded during natural recovery, they remained significantly lower than those in the CON group. In contrast, muscle function in the IM + EX group improved significantly compared with the IM group (Figures S3A and 1A). Immobilization led to atrophy of the posterior calf muscles, whereas combined exercise promoted skeletal muscle hypertrophy (Figure 1B), as indicated by the increased wet weights and body weight ratios of the gastrocnemius and soleus muscles (Figures S3B and 1C). No significant differences were observed in wet weight‐to‐body weight ratios for other muscles and fat tissues (Figure S3C). Another hallmark of muscle atrophy is a reduction in muscle fibre size. As expected, the CSA and diameter of gastrocnemius muscle fibres were significantly reduced following immobilization, whereas exercise exerted a protective effect against this reduction (Figure 1D). The distribution of muscle fibre diameters shifted leftward in the immobilized group and rightward in the exercised group, further supporting this observation (Figure 1E). Additionally, immobilization‐induced muscle atrophy is often accompanied by muscle fibrosis, a primary cause of decreased extensibility and limited range of motion. Exercise mitigated the increase in skeletal muscle collagen content induced by immobilization (Figure 1F).

The primary cause of decreased muscle mass and CSA is an imbalance between protein degradation and synthesis. To investigate this, we examined molecules involved in regulating these processes (Figure S3D). MuRF1 and Atrogin‐1, two muscle‐specific E3 ubiquitin ligases, are upregulated in several models of muscle atrophy and serve as sensitive biomarkers of muscle degradation. Consistent with previous studies [20], exercise reversed the immobilization‐induced upregulation of these proteolysis‐associated proteins. Myosin heavy chain (MyHC), a major component of thick myofilaments crucial for skeletal muscle contraction, was downregulated following immobilization but restored by exercise. Interestingly, the expression of the protein synthesis regulator p‐mTOR and its downstream target p‐S6K1 increased after immobilization and remained elevated in the exercise group. To clarify this observation, we assessed skeletal muscle protein synthesis rates using puromycin incorporation, which revealed no significant differences among the three groups (Figure S3E). Muscle stem cells (MuSCs) play a crucial role in muscle repair and fibre regeneration following injury. We hypothesized that the regenerative capacity of muscle might be impaired during immobilization. To test this hypothesis, we assessed the fluorescence signal of Pax7, a marker for MuSCs, but surprisingly, no significant differences were observed among the three groups (Figure S4A,B). We further analysed several molecules involved in regulating MuSCs proliferation, regeneration and differentiation (Figure S4C). Although Pax7 expression showed a tendency to decrease after immobilization, the levels of the myogenic regulators MyoD1, MyoG and MEF2 remained unchanged across the three groups. These findings suggest that neither immobilization nor combined exercise significantly alters the reserve capacity for muscle regeneration. In summary, our results demonstrate that immobilization leads to an imbalance between muscle protein synthesis and degradation, along with an increase in collagen content. In contrast, exercise improves muscle function and mass, mitigates immobilization‐induced myasthenia and reduces collagen content.

### 3.3 Immobilization Downregulated CSE/H_2_S Expression in Skeletal Muscle and Exercise Reversed This Effect

**FIGURE 2:**
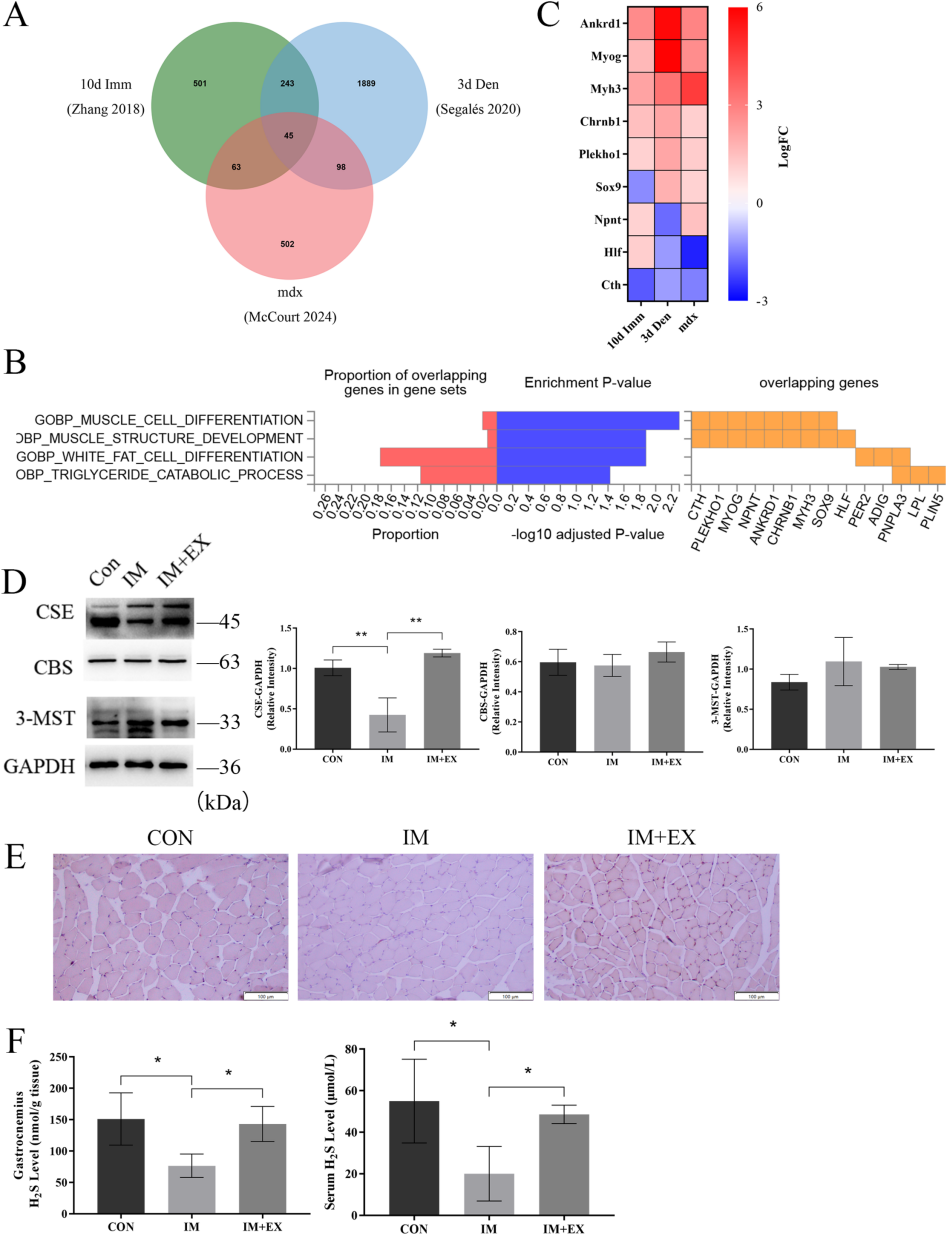
Immobilization downregulated CSE/H_2_S in skeletal muscle, and exercise reversed this change. (A) Venn diagram showing the overlap of dysregulated genes between immobilized (Imm), denervated (Den) and Duchenne muscular dystrophy (mdx) muscles as reported in published muscle atrophy models (see Table S1). (B) Gene Ontology (GO) analysis illustrating the main biological processes enriched in the dysregulated genes. (C) Heatmap depicting aberrant transcription of nine genes involved in muscle‐related biological processes. (D) Western blot analysis of proteins associated with H_2_S production in GAS muscle. (E) Immunohistochemical (IHC) staining for CSE in GAS muscle. Scale bar: 100 μm. (F) Methylene blue assay measuring H_2_S levels in GAS muscle and serum. One‐way ANOVA was used for D (*n* = 3) and F (*n* = 4). Values are means ± SEM. *p < 0.05; **p < 0.01.

We further investigated the mechanisms through which exercise ameliorates muscle atrophy. Bioinformatics analysis of transcriptomes associated with muscle atrophy in models of immobilization, denervation and Duchenne's muscular dystrophy identified 45 genes with dysregulated expression across all datasets (Figure 2A and Table S1). Gene ontology enrichment analysis revealed that these differentially expressed genes were primarily involved in biological processes such as muscle cell differentiation and muscle structure development (Figure 2B). A heatmap analysis of genes associated with these processes demonstrated consistent expression trends across all models, with Cth being downregulated in all three models of muscle atrophy (Figure 2C). Cth encodes CSE, one of the three enzymes responsible for generating H_2_S in mammals, alongside CBS and 3‐MST. We examined the levels of these enzymes in skeletal muscle (Figure 2D). Consistent with transcriptomic data, immobilization reduced the protein expression of CSE, but not CBS or 3‐MST, as shown by CSE immunohistochemical staining (Figure 2E. Previous studies have reported that CSE is the primary enzyme responsible for H_2_S production in mouse skeletal muscle [8]. Additionally, H_2_S levels in the gastrocnemius muscle and serum were decreased in immobilized mice (Figure 2F), indicating that the CSE/H_2_S pathway may play a role in disuse muscle atrophy. Notably, exercise effectively reversed the reduction in CSE and H_2_S levels (Figure 2E,F). Previous studies have shown that exercise training enhances cardiac H_2_S biosynthesis and prevents high‐fat diet‐induced diabetic cardiomyopathy by reducing cell death [13]. In summary, the CSE/H_2_S pathway responds to the protective effects of exercise on muscle atrophy, and we propose that H_2_S may represent a novel gaseous mediator of exercise‐induced benefits.

### 3.4 Skeletal Muscle CSE/H_2_S Played a Protective Role in Disuse Muscle Atrophy

**FIGURE 3:**
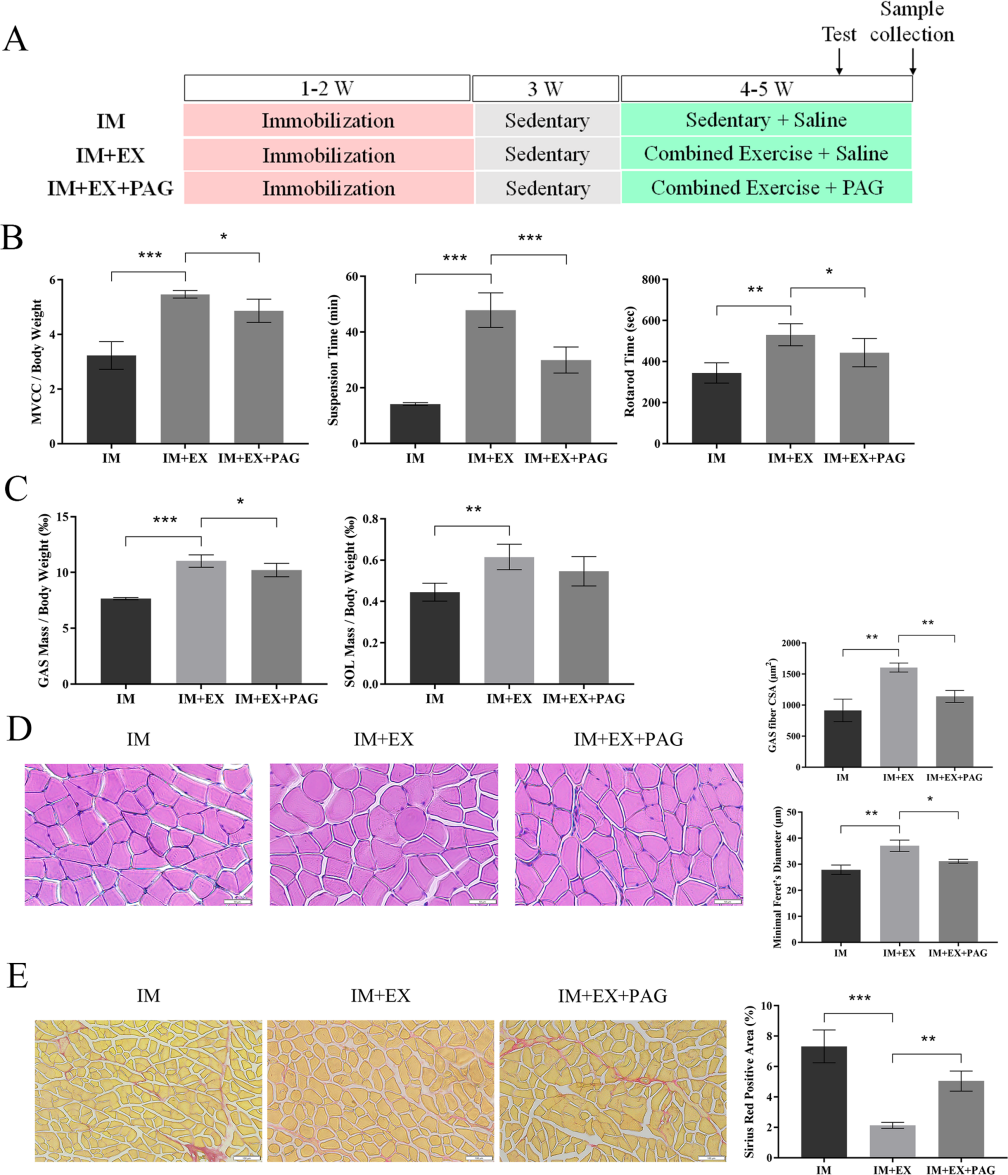
Inhibition of CSE/H_2_S diminished the protective effects of exercise on disuse muscle atrophy. (A) Experimental design. (B) Comparison of exercise capacity between the CSE inhibition group (IM + EX+PAG) and the exercise group (IM + EX). (C) GAS and SOL muscles adjusted for body weight. (D) H&E staining of GAS muscle to assess myofiber CSA and diameter. Scale bar: 50 μm. (E) Sirius red staining of GAS muscle to evaluate collagen deposition. Scale bar: 100 μm. One‐way ANOVA was used for B, C (*n* = 7) and D, E (*n* = 3). Values are means ± SEM *p < 0.05; **p < 0.01; ***p < 0.001. PAG: DL‐propargylglycine.

DL‐Propargylglycine (PAG), an irreversible inhibitor of CSE, suppresses CSE activity through substrate competition, thereby reducing H_2_S production. To investigate whether inhibiting the CSE/H_2_S pathway would attenuate the beneficial effects of exercise, we administered intraperitoneal injections of PAG (50 mg/kg/day) to mice in the IM + EX + PAG group, whereas the remaining two groups received equal volumes of saline (Figure 3A). The efficacy of PAG was confirmed by a significant reduction in H_2_S levels in the gastrocnemius muscle and serum (Figure S5A). PAG treatment during exercise led to a significant decline in muscle strength and locomotor capacity (Figure 3B). Compared with the exercise group, the inhibitor group exhibited a reduction in posterior calf muscle mass (Figure S5B), a reduced ratio of gastrocnemius wet weight to body weight and a tendency towards decreased mass of the soleus muscle (Figure 3C). HE and Sirius red staining revealed significantly smaller CSA and diameters of gastrocnemius muscle fibres in the inhibitor group compared with the exercise group (Figure 3D), along with a marked leftward shift in fibre diameter distribution (Figure S5C) and increased collagen fibre content (Figure 3E). These findings suggest that inhibiting CSE/H_2_S diminishes the protective effects of exercise on disuse muscle atrophy.

**FIGURE 4:**
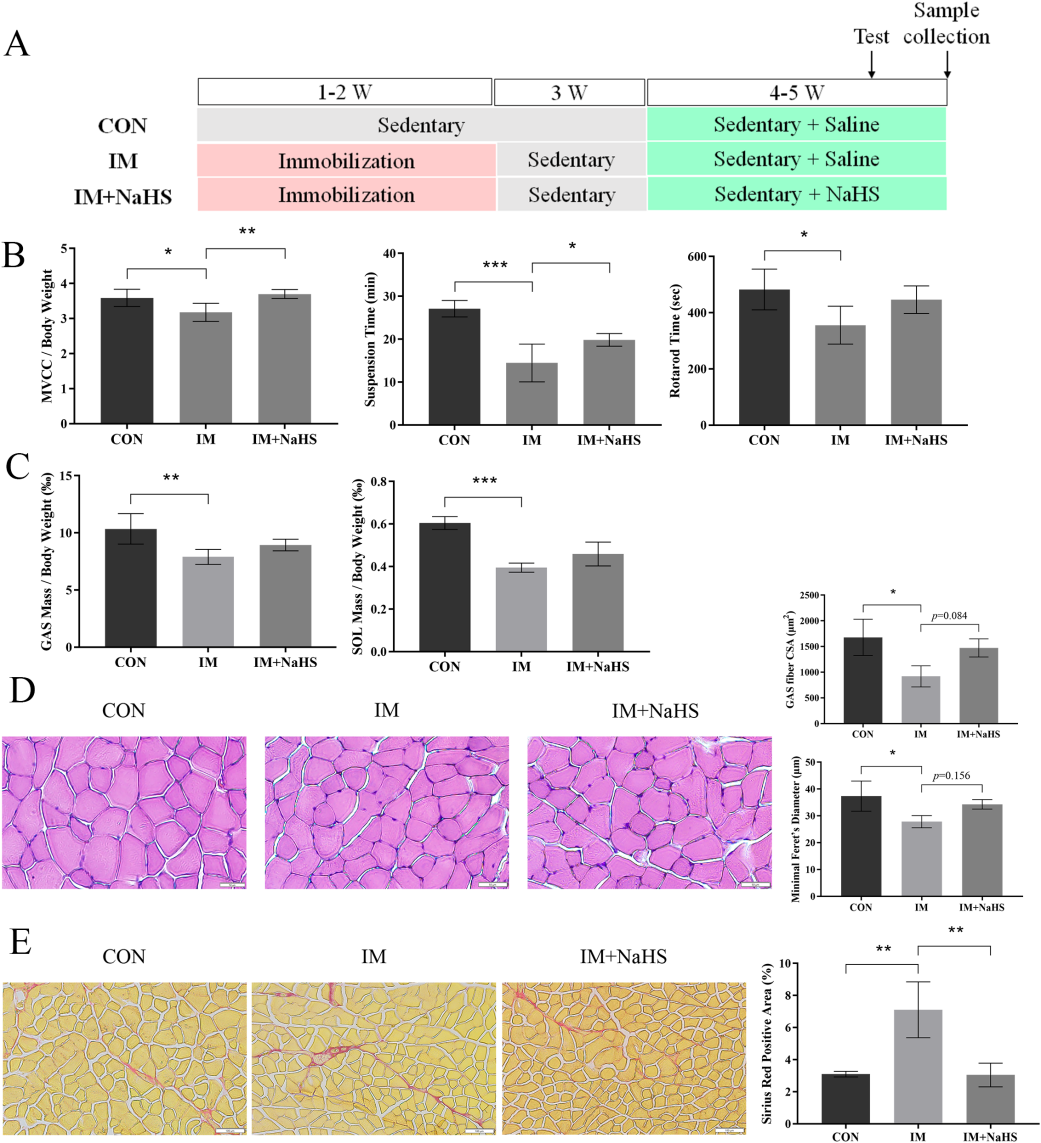
H_2_S donor treatment alleviated disuse muscle atrophy to some extent. (A) Experimental design. (B) Comparison of exercise capacity between the H_2_S‐supplemented group (IM + NaHS) and the immobilization group (IM). (C) GAS and SOL muscles adjusted for body weight. (D) H&E staining of GAS muscle to assess myofiber CSA and diameter. Scale bar: 50 μm. (E) Sirius red staining of GAS muscle to evaluate collagen deposition. Scale bar: 100 μm. One‐way ANOVA was used for B, C (*n* = 7) and D, E (*n* = 3). Values are means ± SEM *p < 0.05; **p < 0.01; ***p < 0.001.

In contrast, we treated mice with disuse muscle atrophy using an exogenous H_2_S donor, sodium hydrosulphide (NaHS) (30 μmol/kg, twice daily) via intraperitoneal injection (Figure 4A). After 2 weeks of treatment, the H_2_S levels in the gastrocnemius muscle and serum of the IM + NaHS group were significantly elevated, confirming the effectiveness of the exogenous H_2_S donor (Figure S5D). NaHS treatment partially restored locomotor abilities, as indicated by significant improvements in MVCC/body weight and suspension time (Figure 4B). NaHS treatment did not significantly increase the mass of the gastrocnemius and soleus muscles (Figure 4C). Still, HE staining analysis revealed a partial recovery in muscle fibre CSA (*p* = 0.084) and fibre diameter (*p* = 0.156) of the gastrocnemius muscle (Figure 4D), with a rightward shift in the distribution of muscle fibre diameters (Figure S5E). Additionally, NaHS treatment significantly reduced collagen fibre content in the gastrocnemius muscle (Figure 4E). These findings suggest that exogenous H_2_S supplementation mitigated disuse muscle atrophy to some extent. Overall, the loss‐of‐function and donor treatment experiments underscore the protective role of CSE/H_2_S against disuse muscle atrophy.

### 3.5 H_2_S Protected Skeletal Muscle From Immobilization‐Induced Oxidative Stress

**FIGURE 5:**
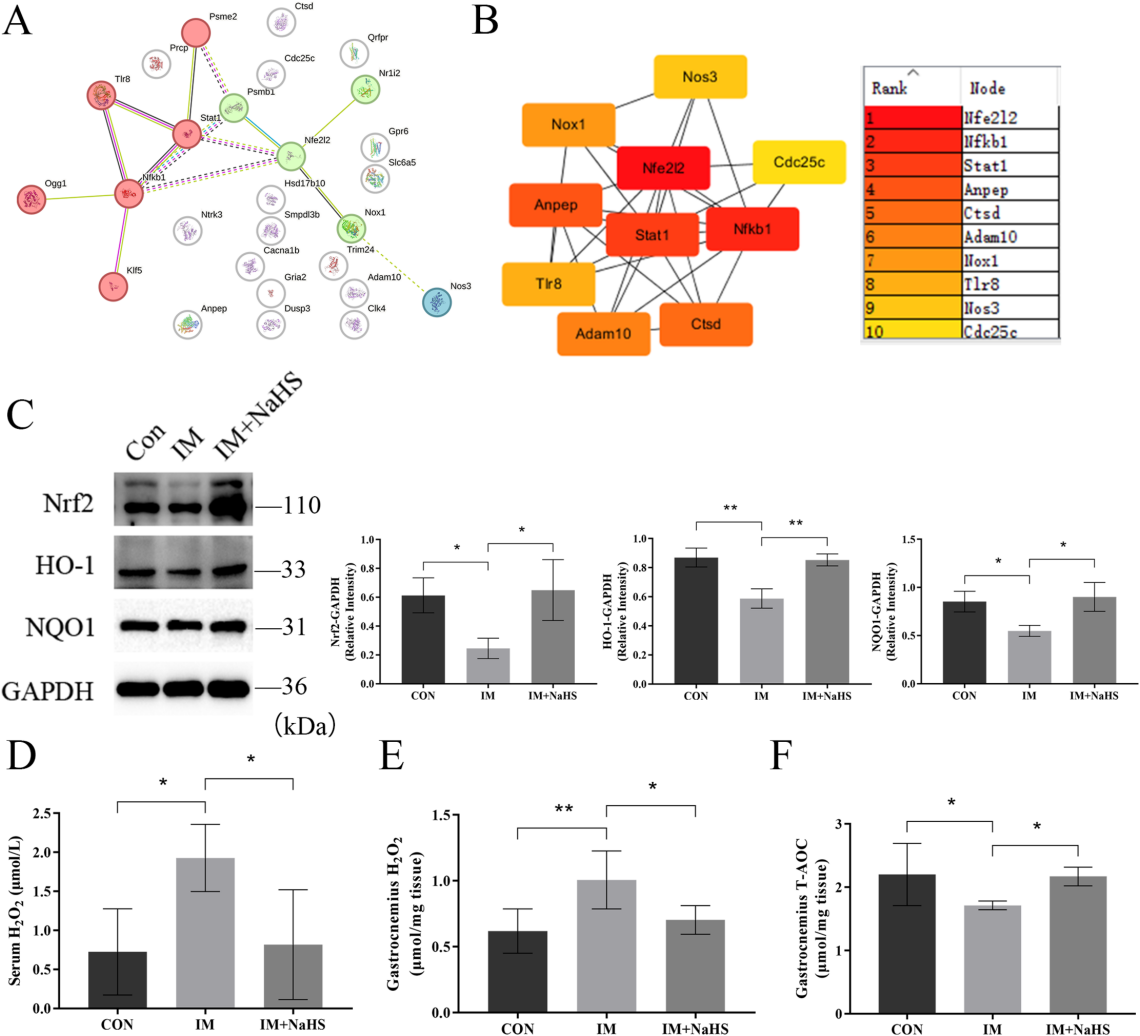
H_2_S protected skeletal muscle from immobilization‐induced oxidative stress via Nrf2. (A) The Super‐PRED database was used to predict the common potential targets of H_2_S donors NaHS and GYY4137 (see Tables S2 and S3). (B) Protein–protein interaction (PPI) networks of potential targets were generated using the STRING database and Cytoscape software to identify key targets (see Table S4). (C) Western blot analysis of oxidative stress‐related protein expression in GAS muscle. (D–E) H_2_O_2_ levels in serum and GAS muscle. (F) Total antioxidant capacity (T‐AOC) assay of GAS muscle. One‐way ANOVA was used for C (*n* = 3) and D–F (*n* = 5–6). Values are means ± SEM. *p < 0.05; **p < 0.01.

To further investigate the mechanisms by which H_2_S alleviates disuse muscle atrophy, we used SuperPred (https://prediction.charite.de/index.php/) to predict potential targets of two commonly used exogenous H_2_S donors, NaHS and GYY4137. A total of 27 intersecting targets were identified (Figure 5A and Tables S2 and S3). These targets were subjected to protein–protein interaction (PPI) analysis using the STRING database (https://cn.string‐db.org/) with a confidence score of 0.15, and the results were visualized using Cytoscape software to identify core targets. The top five core targets identified were Nfe2l2, Nfkb1, Stat1, Anpep and Ctsd (Figure 5B and Table S4). Prolonged muscle immobilization exacerbates oxidative stress through increased production of free radicals and reactive oxygen species (ROS), which are key contributors to disuse muscle atrophy [22], and Nfe2l2 encodes Nrf2, a critical regulator of oxidative stress. Therefore, we focused on the effects of exogenous H_2_S supplementation on skeletal muscle Nrf2 levels and oxidative stress. As anticipated, Nrf2 protein expression was significantly reduced in skeletal muscle following immobilization but was upregulated after NaHS treatment (Figure 5C). Similar trends were observed for HO‐1 and NQO1, which are downstream targets of Nrf2. Likewise, exercise increased the protein levels of Nrf2 and its downstream targets, whereas treatment with the inhibitor PAG reduced their expression (Figure S6A). H_2_O_2_, a common ROS, was also reduced in both serum and gastrocnemius muscle after H_2_S donor treatment (Figure 5D,E). Additionally, NaHS treatment enhanced the total antioxidant capacity (T‐AOC) of skeletal muscle (Figure 5F). These results suggest that H_2_S protects skeletal muscle from immobilization‐induced oxidative stress by upregulating Nrf2 expression.

### 3.6 H_2_S Exerted Antioxidant Effects Through the SESN2‐Nrf2 Pathway

**FIGURE 6:**
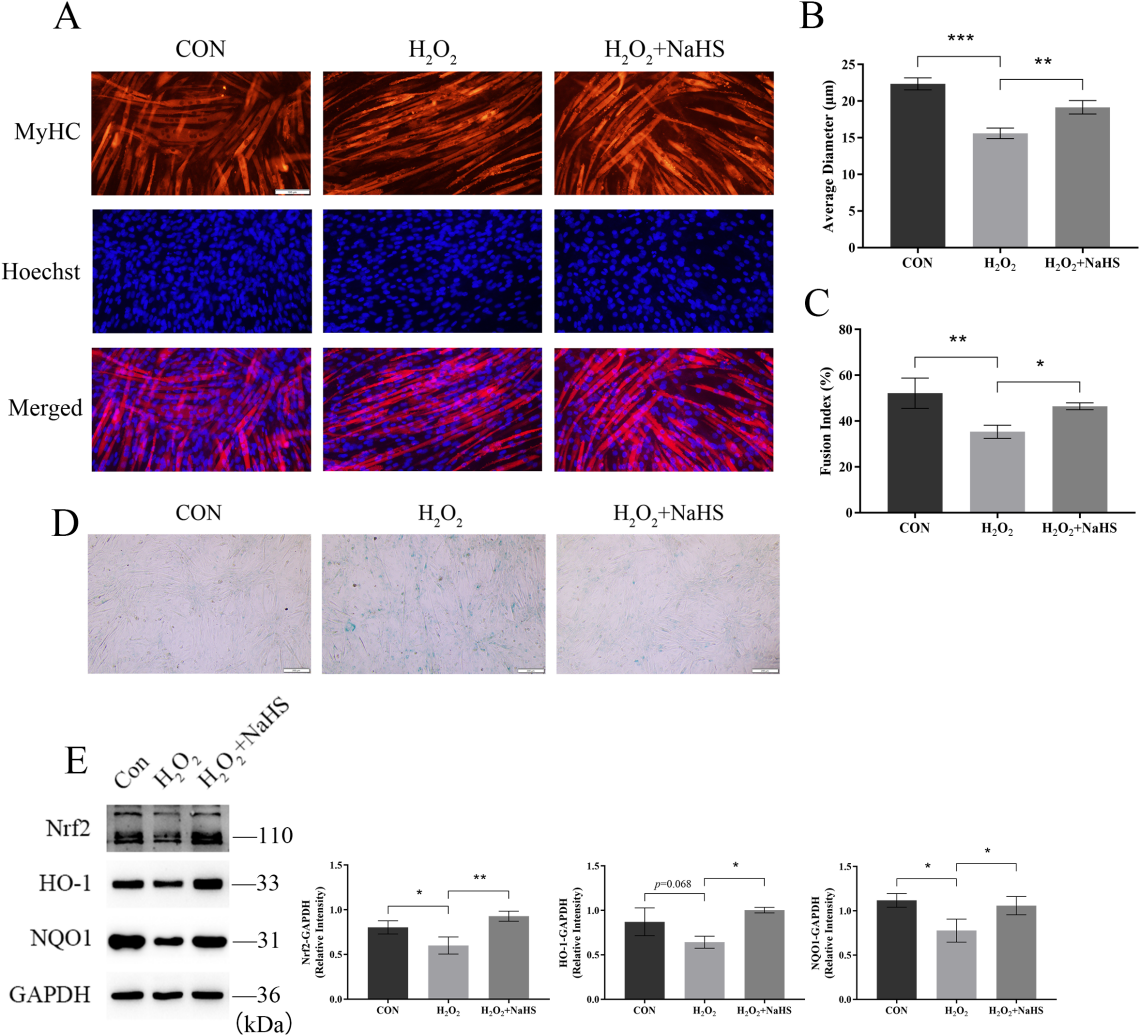
NaHS treatment rescued H_2_O_2_‐induced atrophy in C_2_C_12_ myotubes. (A) Immunofluorescence staining of C_2_C_12_ myotubes showing MyHC (red) and Hoechst (blue). Scale bar: 100 μm. (B) The average diameter of C_2_C_12_ myotubes was analysed using Image J. (C) The myotube fusion index was calculated as the ratio of myotube nuclei to total nuclei. (D) Senescence‐associated β‐galactosidase staining to assess the alleviating effect of NaHS on C_2_C_12_ myotube senescence. Scale bar: 200 μm. (E) Western blot analysis of oxidative stress‐related protein expression in C_2_C_12_ myotubes. One‐way ANOVA was used for B, C and E (*n* = 3). Values are means ± SEM. *p < 0.05; **p < 0.01; ***p < 0.001.

To further confirm that H_2_S mitigates muscle atrophy by modulating oxidative stress, we induced atrophy in C_2_C_12_ myotubes using 0.5‐mM H_2_O_2_, followed by treatment with 30‐μM NaHS. MHC immunofluorescence analysis revealed that H_2_O_2_ significantly reduced the diameter of C_2_C_12_ myotubes and lowered the fusion index, indicating myotube atrophy. These changes were effectively reversed by NaHS treatment (Figure 6A–C). Since H_2_O_2_ is commonly used to induce cellular senescence, we utilized β‐galactosidase staining to detect senescence in C_2_C_12_ cells. As expected, NaHS treatment alleviated H_2_O_2_‐induced senescence (Figure 6D). Furthermore, we examined the proliferation of C_2_C_12_ myogenic cells using the EdU assay, finding that NaHS treatment restored the H_2_O_2_‐induced reduction in proliferative capacity (Figure S6B). Consistent with in vivo results, H_2_O_2_ intervention also downregulated the protein levels of Nrf2 and its downstream targets in C_2_C_12_ cells, whereas NaHS treatment reversed this downregulation (Figure 6E). These findings suggest that NaHS rescues C_2_C_12_ myotubes from oxidative stress‐induced atrophy and enhances myogenic cell viability.

**FIGURE 7:**
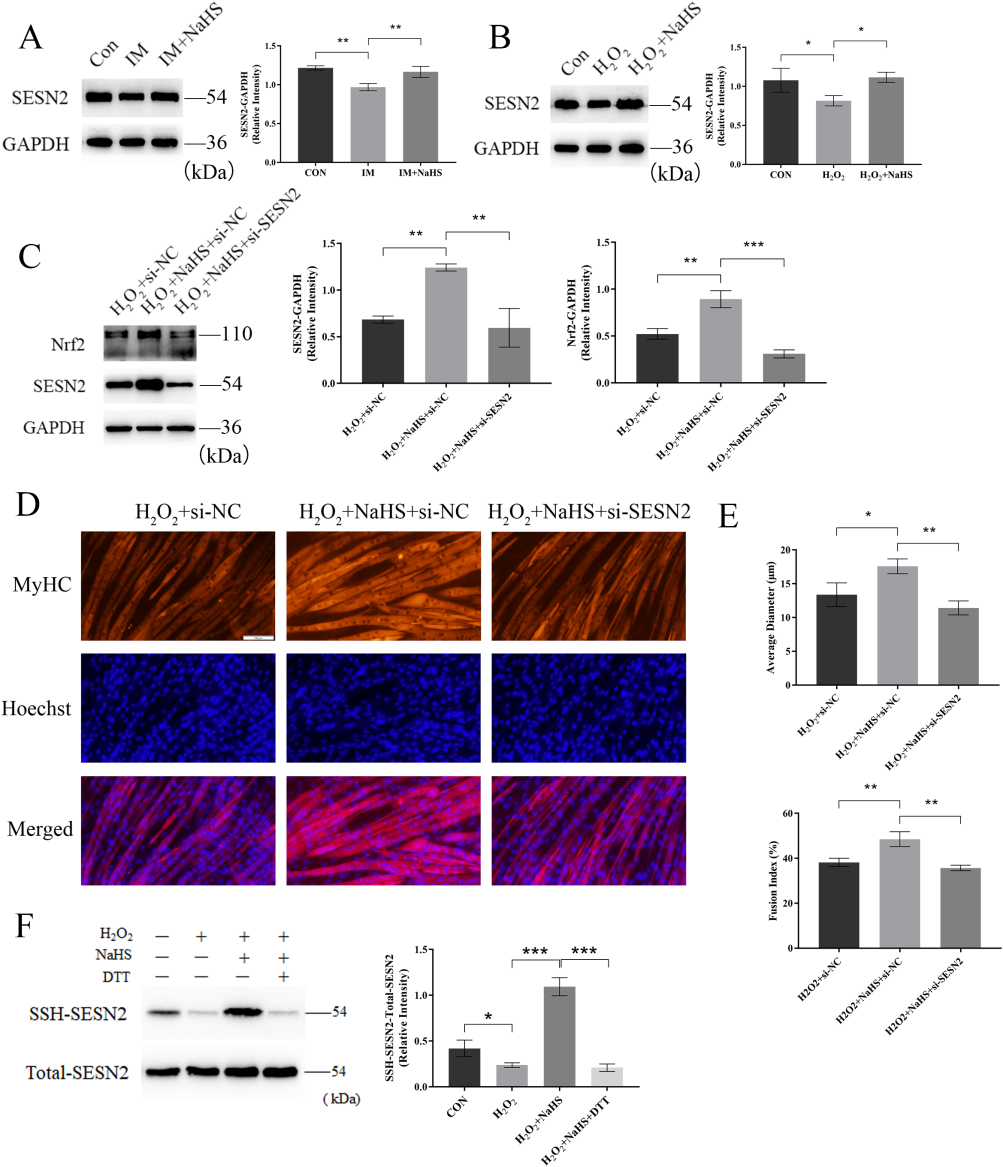
H_2_S exerted its effects via the SESN2‐Nrf2 pathway. (A–B) Western blot analysis showed the impact of NaHS treatment on SESN2 expression in GAS muscle and C_2_C_12_ myotubes. (C) Western blot analysis of SESN2 and Nrf2 expression in C_2_C_12_ myotubes following transfection with SESN2‐siRNA. (D) Immunofluorescence staining of C_2_C_12_ myotubes shows MyHC (red) and Hoechst (blue). Scale bar: 100 μm. (E) SESN2‐siRNA treatment reversed the protective effect of NaHS on C_2_C_12_ myotube atrophy. (F) Western blot analysis showed the impact of NaHS treatment on SSH‐SESN2 expression in C_2_C_12_ myotubes. One‐way ANOVA was used for A–C and E–F (*n* = 3–4). Values are means ± SEM. *p < 0.05; **p < 0.01; ***p < 0.001.

Our previous study identified SESN2 as an exercise‐responsive protein, showing that exercise mitigates high‐fat diet‐induced oxidative stress and chronic inflammation through the SESN2‐Nrf2 pathway [23]. Consistent with these findings, combined exercise reversed the immobilization‐induced reduction in SESN2‐Nrf2 protein expression (Figure S5C,D). Similarly, in vitro and ex vivo experiments revealed that NaHS treatment increased SESN2 protein expression in skeletal muscle and C_2_C_12_ myotubes (Figure 7A,B). Based on this, we hypothesized that the regulatory effects of NaHS treatment were mediated via SESN2. To test this hypothesis, we transfected NaHS‐treated C_2_C_12_ myotubes with SESN2‐siRNA to confirm the role of SESN2. SESN2 protein levels were reduced following SESN2‐siRNA treatment, proving the effectiveness of the silencing tool (Figure 7C). When SESN2 was silenced, the reduction in Nrf2 protein expression was associated with a decrease in C_2_C_12_ myotube diameter and fusion index (Figure 7D,E). Considering the potential post‐translational sulfidation effects of H_2_S, we performed a biotin‐switch assay on C_2_C_12_ myotubes treated with NaHS and observed increased levels of persulfidated SESN2 protein (Figure 7F). These findings suggest that H_2_S may enhance antioxidant defence through the SESN2‐Nrf2 pathway, potentially by stabilizing SESN2 via persulfidation, thereby contributing to its protective role in disuse‐induced muscle atrophy.

**FIGURE 8:**
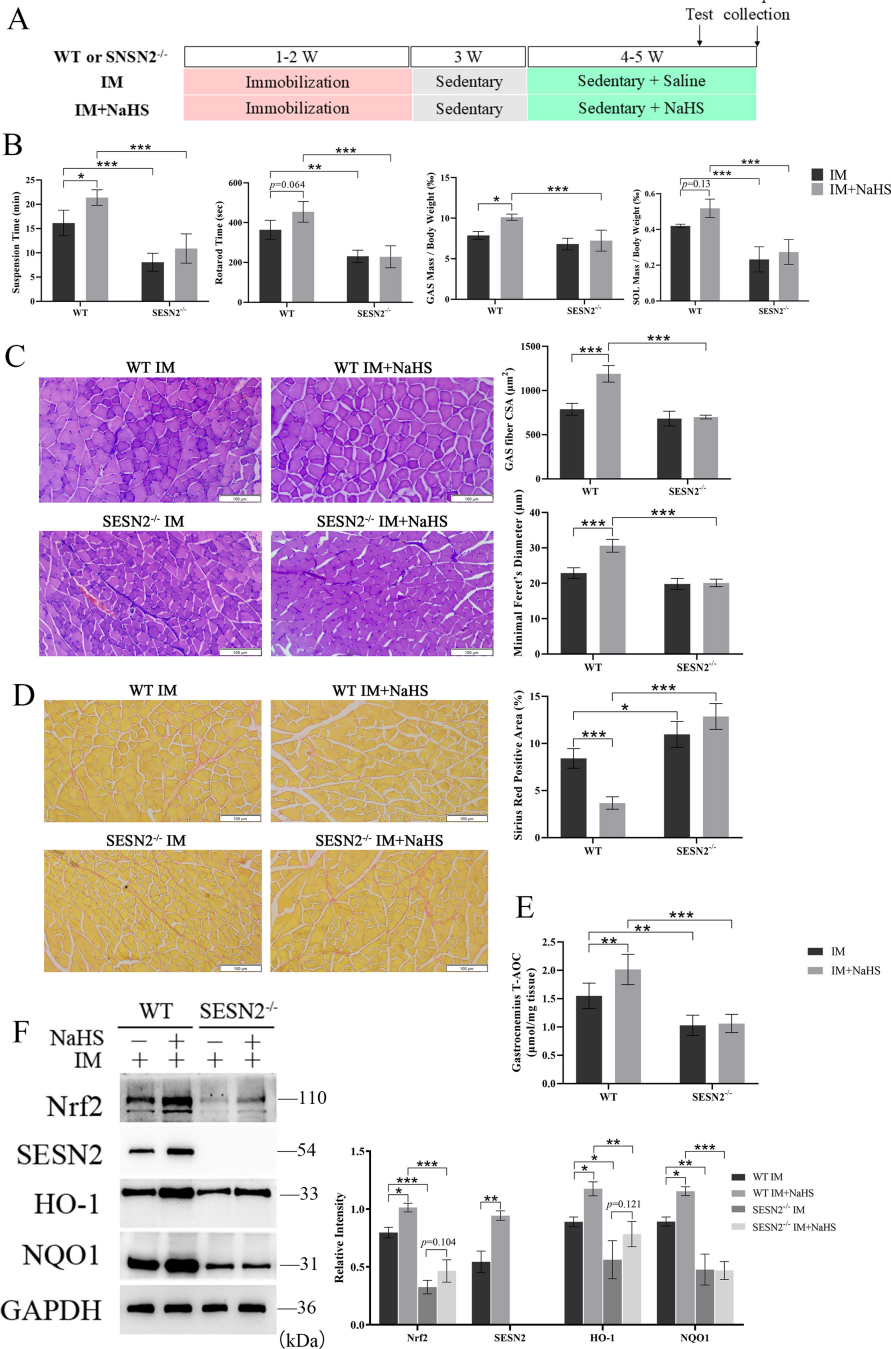
SESN2 knockout abolished the protective effects of H_2_S donors against disuse‐induced muscle atrophy. (A) Experimental design. (B) Comparison of exercise capacity and skeletal muscle mass between SESN2 knockout and wild‐type mice. (C) H&E staining of the gastrocnemius muscle to assess muscle fibre CSA and diameter. Scale bar: 100 μm. (D) Sirius red staining of the gastrocnemius muscle to evaluate collagen deposition. Scale bar: 100 μm. (E) Total antioxidant capacity assay of gastrocnemius muscle. (F) Western blot analysis of SESN2 and oxidative stress‐related protein expression in gastrocnemius muscle. Two‐way ANOVA was used for B (*n* = 4–6), C–E (*n* = 4) and F (*n* = 3). Values are means ± SEM *p < 0.05; **p < 0.01; ***p < 0.001.

To validate the role of the SESN2‐Nrf2 pathway in H_2_S‐mediated antioxidant protection, we generated SESN2 knockout (SESN2^−/−^) mice using the CRISPR/Cas9 system. Littermate SESN2^−/−^ and WT mice were then randomly assigned to either IM or IM + NaHS treatment groups (Figure 8A). Following immobilization, SESN2^−/−^ mice exhibited a more pronounced decline in exercise endurance, motor coordination and skeletal muscle mass, effectively abolishing the protective effects of H_2_S on muscle mass and function (Figure 8B). Additionally, SESN2 knockdown negated the benefits of H_2_S on myofiber CSA, fibre diameter and skeletal muscle fibrosis (Figure 8C,D). Mechanistically, SESN2 deletion impaired the H_2_S‐induced enhancement of skeletal muscle antioxidant capacity (Figure 8E). Furthermore, Western blot analysis revealed that SESN2 knockdown suppressed the activation of Nrf2 and its downstream targets, HO‐1 and NQO1, in skeletal muscle following H_2_S treatment (Figure 8F). Collectively, these findings indicate that H_2_S mitigates disuse muscle atrophy via SESN2‐Nrf2 pathway activation.

## 4 Discussion

This study demonstrates that early exercise intervention improves muscle function, increases muscle mass and reduces fibrosis in immobilization‐induced muscle atrophy. We identified the CSE/H_2_S pathway as a key mediator of these protective effects. CSE inhibition with PAG worsened muscle atrophy, whereas H_2_S donor NaHS mimicked exercise benefits by reducing oxidative stress via SESN2/Nrf2 activation. Six‐month‐old mice were used to model middle‐aged muscle decline, addressing a research gap between acute and aged animal models [4, 8, 10]. This age group better reflects early‐stage muscle deterioration and supports the exploration of timely interventions. Collectively, these findings highlight the therapeutic potential of H_2_S as an exercise‐mimetic agent for preserving muscle mass and function.

Numerous studies have investigated the therapeutic effects of exercise interventions on skeletal muscle atrophy. For instance, 4 weeks of resistance training has been shown to promote sciatic nerve injury repair and improve motor function and muscle atrophy by upregulating GAP‐43 expression [24]. Similarly, 6 weeks of resistance training ameliorates dexamethasone‐induced reductions in muscle mass and endurance capacity, enhancing insulin sensitivity and glucose metabolism [25]. Additionally, 7 days of aerobic exercise preconditioning mitigated changes in mitochondrial dynamics and the activation of atrogenes' expression during the early stages of disuse muscle atrophy, with this protective effect persisting until the seventh day of disuse [26]. Although exercise preconditioning can prevent early muscle atrophy and alleviate mitochondrial dysfunction, its protective effect has temporal limitations, and maintaining this benefit may require higher intensity or longer duration training. In this study, we demonstrated for the first time that early exercise intervention after immobilization has a favourable therapeutic impact on disuse muscle atrophy. This effect is likely associated with the balance between protein synthesis and catabolism, as well as changes in collagen content within skeletal muscle (Figures 1 and S3). These findings may have clinical implications for using exercise therapy in treating muscle atrophy caused by prolonged bed rest, immobilization or ageing.

Previous studies have demonstrated that a deficiency in endogenous H_2_S negatively affects skeletal muscle mass and function. In diabetic animal models, the expression of CSE and H_2_S production was reduced in the skeletal muscle of db/db mice and GK rats, resulting in a significant decrease in muscle mass and myofiber diameter [10, 27]. Gene transcript levels of CSE, CBS and 3‐MST were also reduced in Duchenne muscular dystrophy (DMD), indicating that diminished endogenous H_2_S production may contribute to DMD pathogenesis [28]. In our study, we observed decreased CSE/H_2_S levels in skeletal muscle during disuse‐induced atrophy, without alterations in CBS or 3‐MST expression, corroborating previous findings that CSE is the primary H_2_S‐producing enzyme in mouse skeletal muscle [8]. Notably, we observed for the first time that exercise significantly increased CSE expression and H_2_S production in skeletal muscle (Figure 2). Previous research has also shown that prolonged aerobic exercise elevates endogenous H_2_S production in several organs, including the heart [13], liver [14] and kidneys [29], indicating that H_2_S could be a novel ‘exerkine’ in muscle activity. To clarify the role of the endogenous CSE/H2S system in exercise‐induced amelioration of disuse muscle atrophy, we treated mice in the exercise group with PAG, a CSE‐specific inhibitor. After 2 weeks of PAG administration, the mice exhibited decreased locomotor activity, reduced gastrocnemius muscle mass, smaller muscle fibre CSA and increased collagen deposition. Interestingly, there was no significant reduction in soleus muscle mass, likely due to the muscle fibre composition, as the soleus is predominantly composed of slow‐twitch fibres, whereas the gastrocnemius contains a mix of fast‐ and slow‐twitch fibres. Our study focused on mixed‐fibre muscles such as the gastrocnemius; therefore, caution should be taken when generalizing to predominantly oxidative or glycolytic muscles. Future investigations will incorporate fibre type‐specific analyses of H_2_S enzyme expression to better determine the generalizability of our findings. Another study found that CSE‐deficient (CSE^−/−^) mice exhibited severe age‐related muscle atrophy and cardiotoxin (CTX)–induced muscle damage, likely due to impaired muscle regeneration caused by decreased expression of the myogenic regulator Myogenin [8]. Although a similar myopathic phenotype was observed between CSE^−/−^ mice and our model, we did not find changes in muscle regeneration capacity (Figure S4), which may be due to the activation of MuSCs in response to ageing and muscle injury in CSE^−/−^ mice. Collectively, these findings provide compelling evidence for the systemic modulation of the CSE/H_2_S pathway in the protective effects of exercise against disuse muscle atrophy.

Previous studies have demonstrated that the exogenous H_2_S donor NaHS can mitigate skeletal muscle atrophy induced by type 2 diabetes [27], cardiotoxicity [8] and denervation [30] by reducing inflammation, oxidative stress and protein degradation. However, in our study, treatment of mice with disuse muscle atrophy using NaHS for 2 weeks led to partial recovery of gastrocnemius and soleus muscle mass, as well as an increase in the CSA of gastrocnemius fibres. Notably, collagen deposition was significantly reduced. This outcome may be attributed to the extended duration of immobilization, as our previous study found that the gastrocnemius and soleus muscle mass, along with the CSA of gastrocnemius fibres, decreased more substantially after 14 days of immobilization compared with 7 days [20]. The 2‐week NaHS treatment was not sufficient to completely reverse these effects. Surprisingly, NaHS treatment significantly improved muscle strength and endurance in mice. Consistent with our findings, prior studies have shown that intraperitoneal NaHS injections for 8 weeks improved grip strength in GK diabetic rats [27]. Besides the improved muscle mass, this enhancement in exercise capacity may also be linked to the influence of H_2_S on the neuromuscular junction. It has been shown that NaHS promotes quantal transmitter release from motor nerve endings in mammalian neuromuscular synapses, whereas the H_2_S inhibitors BCA and AOAA had opposing effects, suggesting a stimulatory role for H_2_S in neuromuscular activity [31]. The H_2_S donor NaHS was selected for this study because of its rapid‐release properties, which are effective for investigating the direct mechanistic role of H_2_S in exercise‐mediated muscle protection. However, it is important to note that the transient surge in H_2_S concentration induced by NaHS does not fully replicate the physiological kinetics of endogenous H_2_S, thereby limiting its translational potential. In contrast, natural H_2_S donors, such as glucoraphanin derived from radish, release H_2_S more gradually and maintain stable concentrations that better reflect physiological conditions. These agents have demonstrated prolonged protective effects in preclinical models [32]. In conclusion, our study demonstrates that exogenous H_2_S supplementation alleviates disuse muscle atrophy to some extent.

Oxidative stress has been recognized as a critical factor in various muscle atrophies [22]. Nrf2 plays a pivotal role in the regulation of oxidative stress and has been implicated in the pathogenesis of skeletal muscle atrophy associated with chronic kidney disease [33], Type 1 diabetes mellitus [34] and long‐term glucocorticoid therapy [35]. In this study, using network pharmacology screening, we identified and confirmed Nrf2 as a downstream target of H_2_S. Both in vivo and in vitro results demonstrated that NaHS treatment increased Nrf2 protein expression and its downstream effectors, whereas the CSE inhibitor PAG produced the opposite effect. Our previous study showed that exercise reduces high‐fat diet‐induced oxidative stress and chronic inflammation through SESN2/Nrf2 interaction [23]. Furthermore, SESN2 has been reported to activate Nrf2 by promoting Keap1 degradation, thereby protecting the liver from oxidative damage [36]. Our data suggest that SESN2 may be a target of H_2_S and that SESN2/Nrf2 levels are modulated by NaHS. Conversely, the protective effect of H_2_S donors against H_2_O_2_‐induced myotube atrophy and immobilization‐induced muscle atrophy was abolished by SESN2 knockdown. Thus, this study reveals for the first time that the SESN2/Nrf2 pathway may be a novel molecular mechanism underlying the protective effects of H_2_S on skeletal muscle.

This study demonstrated that the CSE/H_2_S/SESN2 mediates exercise protection against immobilization‐induced muscle atrophy; however, it does not rule out the involvement of additional mechanisms. In this study, only male mice were used to eliminate the variability associated with the oestrous cycle and to maintain consistency with previous research on H_2_S in muscle atrophy. However, it is important to acknowledge that sex‐specific biological differences—such as oestrogen's roles in antioxidant defence and metabolic regulation—may limit the generalizability of these findings. Therefore, future studies should investigate sex‐dependent variations in the CSE/H_2_S signalling pathway and evaluate its therapeutic potential in female models. One limitation of this study is the reliance on a short‐term, 2‐week intervention to evaluate the protective effects of exercise and H_2_S treatment against disuse‐induced muscle atrophy. The long‐term sustainability of these effects remains unclear. Future studies are needed to assess the prolonged efficacy and safety of these interventions. Extended or cyclic treatment paradigms may offer further insights into maintaining muscle mass, potential desensitization or adverse outcomes over time. Although our findings suggested that activation of the CSE/H_2_S pathway confers protection against disuse‐induced muscle atrophy, we acknowledge that the current evidence is predominantly derived from systemic interventions. Notably, skeletal muscle‐specific knockout of Cth under basal conditions does not result in significant muscle atrophy [37]. This implies that CSE/H_2_S may exert its protective effects through both muscle‐intrinsic and systemic pathways, though the precise mechanisms remain to be fully elucidated. Future studies should utilize muscle‐specific AAV‐mediated overexpression or deletion of CSE to clarify its direct role in muscle physiology and pathology. Given the known role of H_2_S in redox‐sensitive protein modification [38, 39] and cys125 of SESN2 has also been implicated in its antioxidant function [40], biotin‐switch assays confirmed that H_2_S promotes the persulfidation of SESN2 in C_2_C_12_ myotubes. Further investigations are warranted to identify the specific modification sites and to advance the development of H_2_S‐based therapies for muscle atrophy.

## Conflicts of Interest

The authors declare no conflicts of interest.

## Supporting information


**Data S1:** Supporting Information.

**Table S1:** Supporting Information.

**Table S2:** Supporting Information.

**Table S3:** Supporting Information.

**Table S4:** Supporting Information.

**Figure S1:** Experimental design and images of limb immobilization and exercise interventions. Panel A illustrates the detailed experimental protocol. Panel B shows posterior limb immobilization using cast. Panels C and D show aerobic and resistance exercise training, respectively.

**Figure S2:** Hindlimb immobilization resulted in weight loss and reduced exercise capacity in mice. (A) Body weight comparison before and after immobilization using a paired two‐tailed *t*‐test (*n* = 7). ***p < 0.001. (B) Daily food intake during the first week of immobilization. (C) Total food intake during the immobilization period. (D) MVCC test and adjusted for body weight. (E) Suspension test. (F) Rotarod test. One‐way ANOVA was used for B–G (*n* = 6). Values are means ± SEM. *p < 0.05; **p < 0.01; ***p < 0.001 vs. CON group.

**Figure S3:** Effects of combined exercise on tissue index and skeletal muscle degradation/synthesis in mice following immobilization. (A) MVCC test. (B) GAS and SOL muscle mass. (C) TA, EDL and QUA muscle and adipose tissue mass adjusted for body weight. (D) Protein degradation and synthesis‐related protein expression were detected by Western blot in GAS muscle. (E) Representative blot and quantification of puromycin incorporation to detect de novo protein synthesis in GAS muscle. One‐way ANOVA was used for A–C (*n* = 6) and D and E (*n* = 3). Values are means ± SEM. *p < 0.05; **p < 0.01; ***p < 0.001. TA: tibialis anterior.

**Figure S4:** Immobilization and combined exercise did not impair muscle regeneration. (A) Immunofluorescence was used to quantify the number of MuSCs in GAS muscle. Representative images show Pax7 staining (green) and Hoechst nuclear staining (blue). Scale bar: 100 μm. (B) Quantitative analysis of Pax7‐positive cells in GAS muscle. (C) Western blot analysis of proteins related to muscle regeneration in GAS muscle. One‐way ANOVA was used for B and C (*n* = 3). Values are means ± SEM. MuSCs: muscle stem cells.

**Figure S5:** Protective effect of CSE/H_2_S in disuse muscle atrophy. (A) Effectiveness of CSE‐specific inhibitor PAG. (B) Representative images of hindlimb morphology and muscles in each group after PAG intervention. (C) Distribution of GAS myofiber diameters following PAG intervention. (D) Effectiveness of the H_2_S donor NaHS. (H) Body weight changes during NaHS intervention. (E) Distribution of GAS myofiber diameters following NaHS intervention. One‐way ANOVA was used for A and D (*n* = 3–4). Values are means ± SEM. *p < 0.05; **p < 0.01.

**Figure S6:** Effects of exercise and PAG intervention on the SESN2‐Nrf2 pathway and restoration of C_2_C_12_ myoblasts viability through NaHS treatment. (A) Western blot analysis showed the impact of PAG intervention on oxidative stress‐related protein expression in GAS muscle. (B) Edu staining to assess the proliferative capacity of C_2_C_12_ myoblasts, with Edu staining (red) and Hoechst nuclear staining (blue). Scale bar: 100 μm. Quantitative analysis of Edu‐positive cell percentage using Image J. (C) Western blot analysis showing the effect of immobilization and exercise interventions on the expression of the SESN family in GAS muscle. (D) Western blot analysis of the effect of immobilization and exercise intervention on oxidative stress‐related protein expression in GAS muscle. One‐way ANOVA was used for A–D (*n* = 3–4). Values are means ± SEM. *p < 0.05; **p < 0.01; ***p < 0.001. Edu: 5‐Ethynyl‐20‐deoxyuridine.

## References

[1] K. Mukund and S. Subramaniam , “Skeletal Muscle: A Review of Molecular Structure and Function, in Health and Disease,” Wiley Interdisciplinary Reviews: Systems Biology and Medicine 12, no. 1 (2020): e1462. 31407867 10.1002/wsbm.1462 PMC6916202

[2] M. H. Edwards , E. M. Dennison , A. Aihie Sayer , R. Fielding , and C. Cooper , “Osteoporosis and Sarcopenia in Older Age,” Bone 80 (2015): 126–130. 25886902 10.1016/j.bone.2015.04.016 PMC4601530

[3] S. Cohen , J. A. Nathan , and A. L. Goldberg , “Muscle Wasting in Disease: Molecular Mechanisms and Promising Therapies,” Nature Reviews. Drug Discovery 14, no. 1 (2015): 58–74. 25549588 10.1038/nrd4467

[4] J. E. Hurst and R. H. Fitts , “Hindlimb Unloading‐Induced Muscle Atrophy and Loss of Function: Protective Effect of Isometric Exercise,” Journal of Applied Physiology (1985) 95, no. 4 (2003): 1405–1417. 10.1152/japplphysiol.00516.2002 12819219

[5] G. R. Adams , F. Haddad , P. W. Bodell , et al., “Combined Isometric, Concentric, and Eccentric Resistance Exercise Prevents Unloading‐Induced Muscle Atrophy in Rats,” Journal of Applied Physiology (1985) 103, no. 5 (2007): 1644–1654. 10.1152/japplphysiol.00669.2007 17872405

[6] L. Li , P. Rose , and P. K. Moore , “Hydrogen Sulfide and Cell Signaling,” Annual Review of Pharmacology and Toxicology 51 (2011): 169–187. 10.1146/annurev-pharmtox-010510-100505 21210746

[7] U. Sen , P. B. Sathnur , S. Kundu , et al., “Increased Endogenous H_2_S Generation by CBS, CSE, and 3MST Gene Therapy Improves *Ex Vivo* Renovascular Relaxation in Hyperhomocysteinemia,” American Journal of Physiology‐Cell Physiology 303, no. 1 (2012): C41–C51. 22517358 10.1152/ajpcell.00398.2011 PMC3404527

[8] Y. Zhang , L. Masters , Y. Wang , et al., “Cystathionine Gamma‐Lyase/H(2) S Signaling Facilitates Myogenesis Under Aging and Injury Condition,” FASEB Journal 35, no. 5 (2021): e21511. 33826201 10.1096/fj.202002675R

[9] M. Xu , X. Liu , P. Bao , Y. J. Wang , J. Lu , and Y. J. Liu , “H(2)S Protects Against Immobilization‐Induced Muscle Atrophy via Reducing Oxidative Stress and Inflammation,” Frontiers in Physiology 13 (2022): 844539. 35464091 10.3389/fphys.2022.844539 PMC9019569

[10] F. Lu , B. Lu , L. Zhang , et al., “Hydrogen Sulphide Ameliorating Skeletal Muscle Atrophy in db/db Mice via Muscle RING Finger 1 S‐Sulfhydration,” Journal of Cellular and Molecular Medicine 24, no. 16 (2020): 9362–9377. 32633463 10.1111/jcmm.15587 PMC7417732

[11] R. Parsanathan and S. K. Jain , “Hydrogen Sulfide Regulates Irisin and Glucose Metabolism in Myotubes and Muscle of HFD‐Fed Diabetic Mice,” Antioxidants 11, no. 7 (2022): 1369. 35883859 10.3390/antiox11071369 PMC9311985

[12] M. Adel , H. R. H. Elsayed , M. El‐Nablaway , et al., “Targeting Hydrogen Sulfide Modulates Dexamethasone‐Induced Muscle Atrophy and Microvascular Rarefaction, Through Inhibition of NOX4 and Induction of MGF,” M2 Macrophages and Endothelial Progenitors Cells 11, no. 16 (2022): 2500. 36010575 10.3390/cells11162500 PMC9406793

[13] S. Kar , H. R. Shahshahan , B. T. Hackfort , et al., “Exercise Training Promotes Cardiac Hydrogen Sulfide Biosynthesis and Mitigates Pyroptosis to Prevent High‐Fat Diet‐Induced Diabetic Cardiomyopathy,” Antioxidants (Basel) 8, no. 12 (2019): 638. 31835893 10.3390/antiox8120638 PMC6943713

[14] B. Wang , J. Zeng , and Q. Gu , “Exercise Restores Bioavailability of Hydrogen Sulfide and Promotes Autophagy Influx in Livers of Mice Fed With High‐Fat Diet,” Canadian Journal of Physiology and Pharmacology 95, no. 6 (2017): 667–674. 28177674 10.1139/cjpp-2016-0611

[15] A. Ho , C. S. Cho , S. Namkoong , U. S. Cho , and J. H. Lee , “Biochemical Basis of Sestrin Physiological Activities,” Trends in Biochemical Sciences 41, no. 7 (2016): 621–632. 27174209 10.1016/j.tibs.2016.04.005 PMC4930368

[16] T. Wang , Y. Niu , S. Liu , et al., “Exercise Improves Glucose Uptake in Murine Myotubes Through the AMPKalpha2‐Mediated Induction of Sestrins,” Biochimica et Biophysica Acta—Molecular Basis of Disease 1864, no. 10 (2018): 3368–3377. 30048751 10.1016/j.bbadis.2018.07.023

[17] X. Han , Y. Yang , S. Liu , Y. Niu , H. Shao , and L. Fu , “Aerobic Exercise Ameliorates Insulin Resistance in C57BL/6 J Mice via Activating Sestrin3,” Biochimica et Biophysica Acta 1869, no. 1 (2023): 166568. 36220588 10.1016/j.bbadis.2022.166568

[18] L. Wang , X. Liu , S. Liu , Y. Niu , and L. Fu , “Sestrin2 Ablation Attenuates the Exercise‐Induced Browning of White Adipose Tissue in C57BL/6J Mice,” Acta Physiologica 234, no. 3 (2022): e13785. 34995401 10.1111/apha.13785

[19] Y. Yang , X. Yang , Y. Huang , S. Liu , Y. Niu , and L. Fu , “Resistance Exercise Alleviates Dexamethasone‐Induced Muscle Atrophy via Sestrin2/MSTN Pathway in C57BL/6J Mice,” Experimental Cell Research 432, no. 1 (2023): 113779. 37709247 10.1016/j.yexcr.2023.113779

[20] Y. Huang , C. Jiang , X. Li , S. Liu , Y. Niu , and L. Fu , “Resistance Exercise Preconditioning Prevents Disuse Muscle Atrophy by Inhibiting Apoptosis and Protein Degradation via SESN2 in C57BL/6J Mice,” Biochimica et Biophysica Acta 1870, no. 4 (2024): 167111. 38432454 10.1016/j.bbadis.2024.167111

[21] T. Snijders , B. T. Wall , M. L. Dirks , et al., “Muscle Disuse Atrophy Is Not Accompanied by Changes in Skeletal Muscle Satellite Cell Content,” Clinical Science 126, no. 8 (2014): 557–566. 24215591 10.1042/CS20130295

[22] H. Zhang , G. Qi , K. Wang , et al., “Oxidative Stress: Roles in Skeletal Muscle Atrophy,” Biochemical Pharmacology 214 (2023): 115664. 37331636 10.1016/j.bcp.2023.115664

[23] S. Liu , H. Li , Y. Zhang , H. Song , and L. Fu , “Exercise Ameliorates Chronic Inflammatory Response Induced by High‐Fat Diet via Sestrin2 in an Nrf2‐Dependent Manner,” Biochimica et Biophysica Acta 1869, no. 7 (2023): 166792. 37336368 10.1016/j.bbadis.2023.166792

[24] E. Arabzadeh , A. Rahimi , M. Zargani , et al., “Resistance Exercise Promotes Functional Test via Sciatic Nerve Regeneration, and Muscle Atrophy Improvement Through GAP‐43 Regulation in Animal Model of Traumatic Nerve Injuries,” Neuroscience Letters 787 (2022): 136812. 35872241 10.1016/j.neulet.2022.136812

[25] J. Jang , J. H. Koh , Y. Kim , et al., “Balanced Free Essential Amino Acids and Resistance Exercise Training Synergistically Improve Dexamethasone‐Induced Impairments in Muscle Strength, Endurance, and Insulin Sensitivity in Mice,” International Journal of Molecular Sciences 23, no. 17 (2022): 9735. 36077132 10.3390/ijms23179735 PMC9456044

[26] L. Brocca , M. Rossi , M. Canepari , R. Bottinelli , and M. A. Pellegrino , “Exercise Preconditioning Blunts Early Atrogenes Expression and Atrophy in Gastrocnemius Muscle of Hindlimb Unloaded Mice,” International Journal of Molecular Sciences 23, no. 1 (2021): 148. 35008572 10.3390/ijms23010148 PMC8745338

[27] M. S. Bitar , J. Nader , W. Al‐Ali , et al., “Hydrogen Sulfide Donor NaHS Improves Metabolism and Reduces Muscle Atrophy in Type 2 Diabetes: Implication for Understanding Sarcopenic Pathophysiology,” Oxidative Medicine and Cellular Longevity 2018 (2018): 6825452. 30510624 10.1155/2018/6825452 PMC6232794

[28] E. Panza , V. Vellecco , F. A. Iannotti , et al., “Duchenne's Muscular Dystrophy Involves a Defective Transsulfuration Pathway Activity,” Redox Biology 45 (2021): 102040. 34174560 10.1016/j.redox.2021.102040 PMC8246642

[29] B. Seifi , A. Sajedizadeh , M. Kadkhodaee , and M. Ranjbaran , “Long‐Term Exercise Restores Hydrogen Sulfide in the Kidney and Contributes to Exercise Benefits in 5/6 Nephrectomized Rats,” Clinical and Experimental Hypertension 41, no. 1 (2019): 87–91. 29521543 10.1080/10641963.2018.1445752

[30] Y. T. Kuo , P. H. Shih , S. H. Kao , et al., “Pyrroloquinoline Quinone Resists Denervation‐Induced Skeletal Muscle Atrophy by Activating PGC‐1alpha and Integrating Mitochondrial Electron Transport Chain Complexes,” PLoS ONE 10, no. 12 (2015): e0143600. 26646764 10.1371/journal.pone.0143600 PMC4672922

[31] E. Gerasimova , J. Lebedeva , A. Yakovlev , A. Zefirov , R. Giniatullin , and G. Sitdikova , “Mechanisms of Hydrogen Sulfide (H2S) Action on Synaptic Transmission at the Mouse Neuromuscular Junction,” Neuroscience 303 (2015): 577–585. 26192092 10.1016/j.neuroscience.2015.07.036

[32] L. Micheli , E. Mitidieri , C. Turnaturi , et al., “Beneficial Effect of H(2)S‐Releasing Molecules in an In Vitro Model of Sarcopenia: Relevance of Glucoraphanin,” International Journal of Molecular Sciences 23, no. 11 (2022): 5955. 35682634 10.3390/ijms23115955 PMC9180606

[33] M. Huang , Y. Yan , Z. Deng , et al., “Saikosaponin A and D Attenuate Skeletal Muscle Atrophy in Chronic Kidney Disease by Reducing Oxidative Stress Through Activation of PI3K/AKT/Nrf2 Pathway,” Phytomedicine 114 (2023): 154766. 37002971 10.1016/j.phymed.2023.154766

[34] Q. Ding , B. Sun , M. Wang , et al., “N‐Acetylcysteine Alleviates Oxidative Stress and Apoptosis and Prevents Skeletal Muscle Atrophy in Type 1 Diabetes Mellitus Through the NRF2/HO‐1 Pathway,” Life Sciences 329 (2023): 121975. 37495077 10.1016/j.lfs.2023.121975

[35] F. H. Rizk , N. A. Soliman , S. M. Kashef , et al., “Lipoxin A4 Attenuated Dexamethasone‐Induced Muscle Atrophy via Activation of PGC‐1alpha/Nrf2/TFAM Pathway,” Journal of Physiology and Biochemistry 79, no. 1 (2023): 107–115. 36125698 10.1007/s13105-022-00925-1 PMC9905194

[36] S. H. Bae , S. H. Sung , S. Y. Oh , et al., “Sestrins Activate Nrf2 by Promoting p62‐Dependent Autophagic Degradation of Keap1 and Prevent Oxidative Liver Damage,” Cell Metabolism 17, no. 1 (2013): 73–84. 23274085 10.1016/j.cmet.2012.12.002

[37] M. M. Xu , X. G. Liu , P. Bao , et al., “Skeletal Muscle CSE Deficiency Leads to Insulin Resistance in Mice,” Antioxidants 11, no. 11 (2022): 2216. 36358588 10.3390/antiox11112216 PMC9687043

[38] G. Yang , K. Zhao , Y. Ju , et al., “Hydrogen Sulfide Protects Against Cellular Senescence via S‐Sulfhydration of Keap1 and Activation of Nrf2,” Antioxidants & Redox Signaling 18, no. 15 (2013): 1906–1919. 23176571 10.1089/ars.2012.4645

[39] L. Xie , Y. Gu , M. Wen , et al., “Hydrogen Sulfide Induces Keap1 S‐Sulfhydration and Suppresses Diabetes‐Accelerated Atherosclerosis via Nrf2 Activation,” Diabetes 65, no. 10 (2016): 3171–3184. 27335232 10.2337/db16-0020 PMC8928786

[40] H. Kim , S. An , S. H. Ro , et al., “Janus‐Faced Sestrin2 Controls ROS and mTOR Signalling Through Two Separate Functional Domains,” Nature Communications 6 (2015): 10025. 10.1038/ncomms10025 PMC4674687 26612684

