# Supplementary material for: CSE/H2S/SESN2 Signalling Mediates the Protective Effect of Exercise Against Immobilization‐Induced Muscle Atrophy in Mice
Source: J Cachexia Sarcopenia Muscle. 2025 Oct 1;16(5):e70083. doi: 10.1002/jcsm.70083 (PMC12485283; doi:10.1002/jcsm.70083)
Supplement: Supplementary file 1 — Data S1: Supporting Information. [file JCSM-16-e70083-s002.docx]

1.1 Exercise protocol

Briefly, the aerobic exercise involved treadmill running at the intensity of 75% VO_2_max (12m/min) for 30 minutes, 5 days per week. Resistant exercise consisted of climbing a 1-meter ladder (0.02-meter grid, inclined at 85°) for 10 repetitions, 5 days per week. Weights equivalent to 50% of the mouse’s body weight were initially attached to tails and increased by 3g daily. The combined exercise protocol included resistant exercise followed by a 15-minute rest, then aerobic exercise. The training lasted for two weeks.

1.2 H_2_S inhibitor and donor administration

To investigate the impact of CSE/H_2_S mediated exercise on disuse muscle atrophy, mice were administered intraperitoneal injections of DL-Propargylglycine (PAG), an irreversible inhibitor of CSE, at a dose of 50mg/kg once daily during the exercise intervention (IM+EX+PAG). To assess the effect of NaHS, a rapid H_2_S releasing donor, mice were injected intraperitoneally with NaHS at a dose of 30μmol/kg twice per day for two weeks following immobilization (IM+NaHS).

1.3 Muscle function measurement

In the maximal voluntary carrying capacity (MVCC) test, a load equal to 75% of the mouse's body weight was attached to tail, and the load was gradually increased. The maximal weight was recorded when the mouse failed to complete the climb three consecutive times. For the inverted screen test, mice were placed on a wire mesh screen, which was then inverted and held 30 cm above the ground. The time until the mouse fell from the screen was recorded. In the rotarod test, mice underwent two days of adaptive training on a RotaRod System. After training, the rotating rod was accelerated from 5 rpm to 40 rpm over a 5-minute period, and the time to fall was recorded.

1.4 Histology, immunohistochemistry and immunofluorescence

Fresh gastrocnemius tissues were fixed in 4% paraformaldehyde for 48 hours, dehydrated in 30% sucrose, embedded in an optimal cutting temperature (OCT) compound, sectioned into 10μm slices, and stained with hematoxylin or eosin (H*&*E) and Sirius red.

For immunohistochemistry, cryosections were fixed in pre-cooled acetone for 10 minutes, rinsed three times in PBST for five minutes each, and immersed in 3% hydrogen peroxide to inactivate endogenous catalase. Sections were blocked with 0.1% Triton X-100 and 3% BSA in PBS for 30 minutes at room temperature, followed by overnight incubation with anti-CSE (sc-374249, Santa Cruz, 1:200) at 4°C. The next day, slides were incubated with HRP-conjugated secondary antibody for 50 minutes at room temperature, and DAB substrate (P0202, Beyotime) was applied for 3 minutes following the manufacturer's instructions.

For immunofluorescence, cryosections were fixed in 4% paraformaldehyde for 15 minutes and then incubated in a permeabilization and blocking buffer for 1 hour. The sections were then incubated overnight at 4°C with a primary anti-Pax7 antibody (ab187339, Abcam, 1:50), followed by incubation with a fluorophore-conjugated secondary antibody for 1 hour. Nuclei were stained with Hoechst for 10 minutes.

1.5 H_2_S levels measurement

H_2_S levels in gastrocnemius tissues and serum were quantified using a dedicated H_2_S content kit (Solarbio, BC2055). The H₂S content was calculated by measuring the absorbance at 665 nm and comparing it to a standard curve provided by the manufacturer.

1.6 H_2_O_2_ levels measurement

H_2_O_2_ levels were measured using kits (Solarbio, BC3595) according to the manufacturer’s instructions. Gastrocnemius tissue extracts or serum samples were pretreated with excess catalase to eliminate H_2_O_2_, and an H_2_O_2_ standard solution was used as a control to determine the H_2_O_2_ concentration.

1.7 Protein synthesis measurement

Animals were treated with a brief pulse of puromycin. The puromycin-labeled peptides can subsequently be detected using an anti-puromycin antibody. The total amount of puromycin incorporation, as detected by the anti-puromycin antibody (MABE343, MilliporeSigma, 1:8000), reflects the rate of protein synthesis directly. In vivo protein synthesis was assessed by intraperitoneal injection of puromycin (ST551, Beyotime) at a dose of 0.04μmol/g, administered 30 minutes before the collection of gastrocnemius tissue. Protein lysates from the gastrocnemius were then prepared for Western blot analysis.

1.8 Total antioxidant capacity (T-AOC) measurement

T-AOC in gastrocnemius tissue homogenates was measured according to the manufacturer’s instructions (Solarbio, BC1315). The reduction of tripyridyltriazine-Fe^3+^ (TPTZ-Fe^3+^) to tripyridyltriazine-Fe^2+^ (TPTZ-Fe^2+^) under acidic conditions reflects the sample’s total antioxidant capacity. Absorbance was measured at 593 nm.

1.9 Cell culture and treatments

C_2_C_12_ myoblasts were cultured in Dulbecco's Modified Eagle Medium (DMEM) supplemented with 10% fetal bovine serum (FBS) at 37°C with 5% CO_2_. For cell proliferation assays, C_2_C_12_ myoblasts were exposed to 0.5mM H_2_O_2_ for 24 hours, followed by treatment with 30μM NaHS for an additional 24 hours. For differentiation, when the C_2_C_12_ myoblasts reached approximately 80%–90% confluence, cells were cultured in DMEM supplemented with 2% horse serum for 48 hours. The differentiated myotubes were then exposed to 0.5mM H_2_O_2_ for 48 hours and treated with 30μM NaHS for another 48 hours. SESN2 knockdown was achieved through Lipofectamine RNAi-MAX-mediated transfection with SESN2-siRNA during NaHS treatment, while scrambled siRNA was transfected into C_2_C_12_ myotubes as a negative control. H₂O₂ and NaHS concentrations and exposure durations refer to DOI: 10.3389/fnut.2023.1120748; DOI: 10.1096/fj.202002675R.

1.10 5-Ethynyl-20-deoxyuridine (EdU) incorporation assay

An EdU assay was conducted to assess the proliferation of C_2_C_12_ myoblasts using the EdU Cell Proliferation Kit (C0075S, Beyotime). After 48 hours of cultivation, cells were incubated with 50μM EdU solution for 2-hour and fixed with 4% paraformaldehyde and stained with Hoechst.

1.11 Myotubes Immunofluorescence

C_2_C_12_ myotubes were fixed with 4% paraformaldehyde for 20 minutes at room temperature, permeabilized with PBS containing 0.5% Triton X-100 for 30 minutes, and blocked with 5% bovine serum albumin (BSA) for 2 hours. The cells were incubated overnight at 4°C with the primary anti-MyHC antibody (MF-20, DSHB, 2–5μg/ml). Afterward, they were incubated with Alexa Fluor 546 goat anti-mouse IgG (A-11003, Invitrogen, 1:200) for 2 hours and stained with Hoechst for 10 minutes in the dark at room temperature. Myotube diameter was measured by determining the maximum diameter of each myotube using ImageJ software, and the myotube fusion index was calculated by dividing the number of nuclei in myotubes by the total number of nuclei.

1.12 Senescence associated β-galactosidase (SA-β-gal) staining

C_2_C_12_ myotubes were washed with PBS and fixed in staining fixative for 15 minutes at room temperature. The fixed cells were stained with fresh SA-β-gal staining solution at 37°C overnight, following the manufacturer's protocol (Solarbio, G1580). Senescent cells were identified by blue staining.

1.13 Western blot

Protein concentrations extracted from gastrocnemius muscle and C_2_C_12_ cells were measured using the BCA assay. Equal concentrations of proteins were separated by SDS-PAGE at 120 V and transferred to PVDF membranes. The membranes were blocked with 5% skim milk for 1 hour at room temperature, followed by incubation with primary antibodies overnight at 4°C. The primary antibodies used were against MHC (DSHB, MF-20), mTOR (Cell Signaling Technology, #4517), p-mTOR-Ser248 (Cell Signaling Technology, #2974T), p70S6K (Cell Signaling Technology, #9202), p-p70S6K-Thr389 (Cell Signaling Technology, #9205), Atrogin-1 (Proteintech, 67172-1-AP), MuRF1 (Proteintech, 55456-1-AP), Pax7 (Abcam, ab187339), MyoD1 (Proteintech, 18943-1-AP), MyoG (Proteintech, 16048-1-AP), MEF2A (Proteintech, 12382-1-AP), CSE (Santa Cruz, sc-374249), CBS (Santa Cruz, sc-133154), 3-MST (Santa Cruz, sc-376168), Nrf2 (Proteintech, 16396-1-AP), HO-1 (ABclonal, A11102), NQO1 (Abmart, T56710), SESN1 (Abcam, ab134091), SESN2 (Abcam, ab178518), SESN3 (Proteintech, 11431-2-AP), and GAPDH (Utibody, UM4002).Protein bands were visualized using an ECL detection reagent (Millipore) and quantified by densitometry using ImageJ software.

**1.14 Persulfidation by biotin switch assay**

**Persulfidation were detected using a previously described biotin switch method (DOI:10.1016/j.jare.2020.06.023). C_2_C_12_ myotubes lysates were incubated with blocking buffer (PBS containing 20mM methyl methanethiosulfonate [MMTS] and 2.5% SDS) at 50°C for 20 minutes to block free thiol groups. Proteins were then precipitated with four volumes of ice-cold ethanol and incubated at −20°C for 2 hours. The precipitate was collected by centrifugation (5,000 × g, 10 minutes, 4°C) and resuspended in lysis buffer. To label persulfidated cysteine residues, 50mM iodoacetyl–PEG2–biotin was added to the lysis buffer, followed by overnight incubation at 4°C with gentle rotation. Streptavidin-agarose beads were then used to pull down biotinylated proteins. The bound proteins were eluted by boiling in SDS sample buffer and separated via SDS-PAGE. Persulfidation of SESN2 was subsequently detected by Western blot. A negative control was included by adding 1 mM DTT during the biotinylation step to confirm the specificity of persulfidation detection.**

**1.15** Statistical analysis

Experimental data are presented as mean ± SEM. Statistical comparisons between two groups were conducted using unpaired Student’s t-tests. For comparisons involving three or more groups, one-way analysis of variance (ANOVA) followed by LSD post hoc test was applied. Two-way ANOVA was used for analyses involving two independent factors. A *p*-value of less than 0.05 was considered statistically significant. All statistical analyses were performed using GraphPad Prism software.
