# Supplementary figures and images for: CSE/H2S/SESN2 Signalling Mediates the Protective Effect of Exercise Against Immobilization‐Induced Muscle Atrophy in Mice
Source: J Cachexia Sarcopenia Muscle. 2025 Oct 1;16(5):e70083. doi: 10.1002/jcsm.70083 (PMC12485283; doi:10.1002/jcsm.70083)

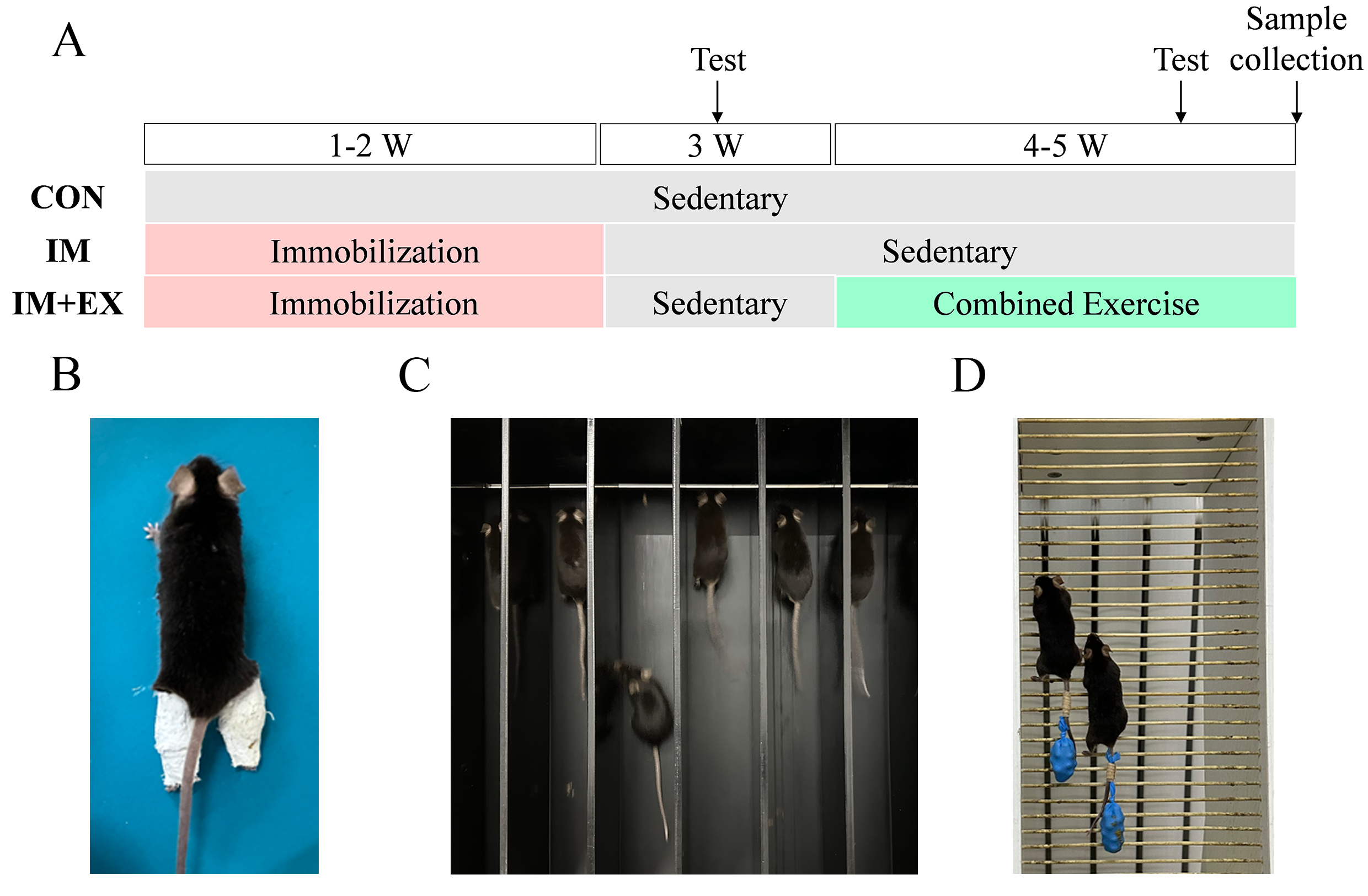

Supplement: Supplementary file 6 — Figure S1: Experimental design and images of limb immobilization and exercise interventions. Panel A illustrates the detailed experimental protocol. Panel B shows posterior limb immobilization using cast. Panels C and D show aerobic and resistance exercise training, respectively. [file JCSM-16-e70083-s007.tif]

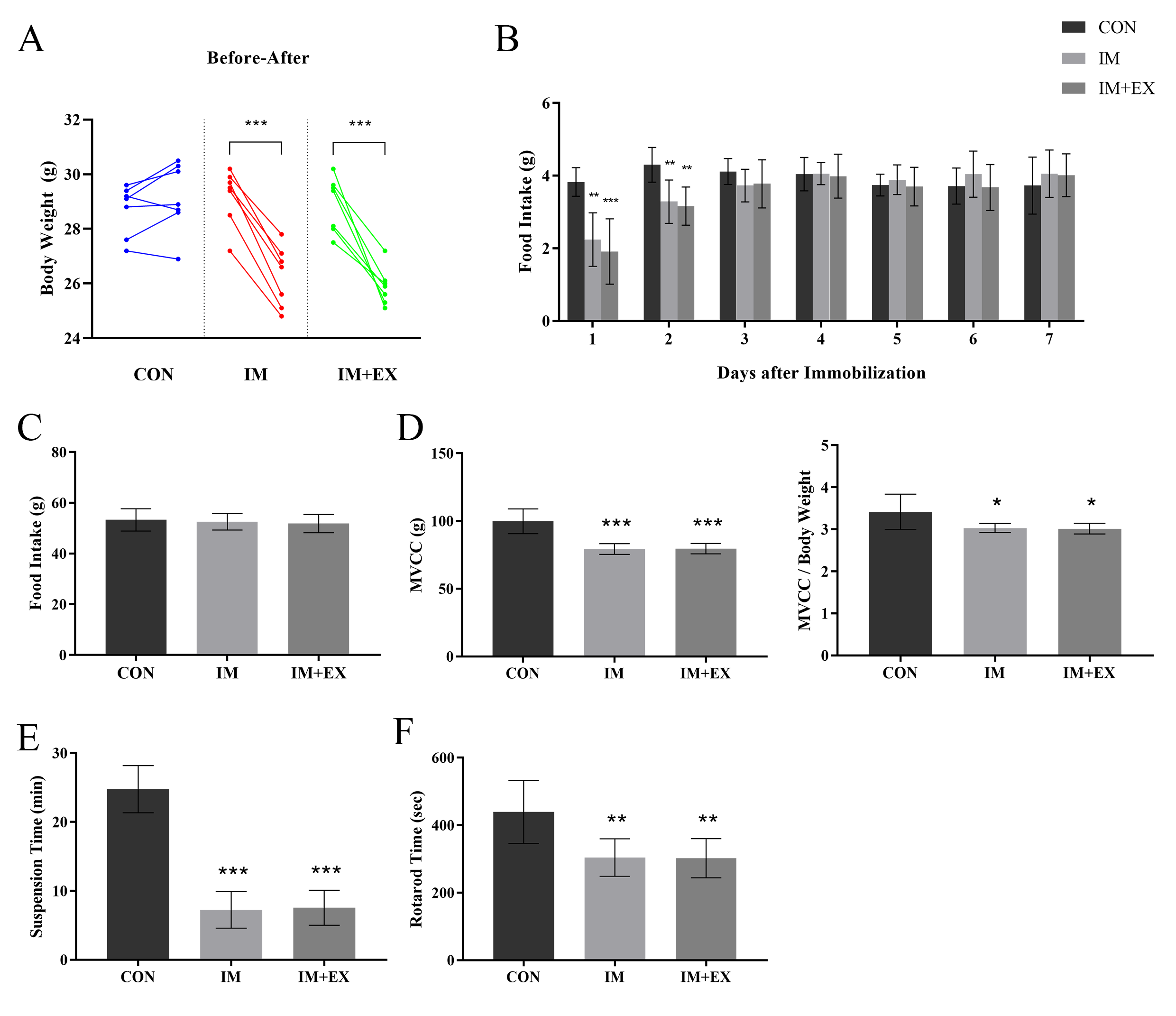

Supplement: Supplementary file 7 — Figure S2: Hindlimb immobilization resulted in weight loss and reduced exercise capacity in mice. (A) Body weight comparison before and after immobilization using a paired two‐tailed t‐test (n = 7). ***p < 0.001. (B) Daily food intake during the first week of immobilization. (C) Total food intake during the immobilization period. (D) MVCC test and adjusted for body weight. (E) Suspension test. (F) Rotarod test. One‐way ANOVA was used for B–G (n = 6). Values are means ± SEM. *p < 0.05; **p < 0.01; ***p < 0.001 vs. CON group. [file JCSM-16-e70083-s011.tif]

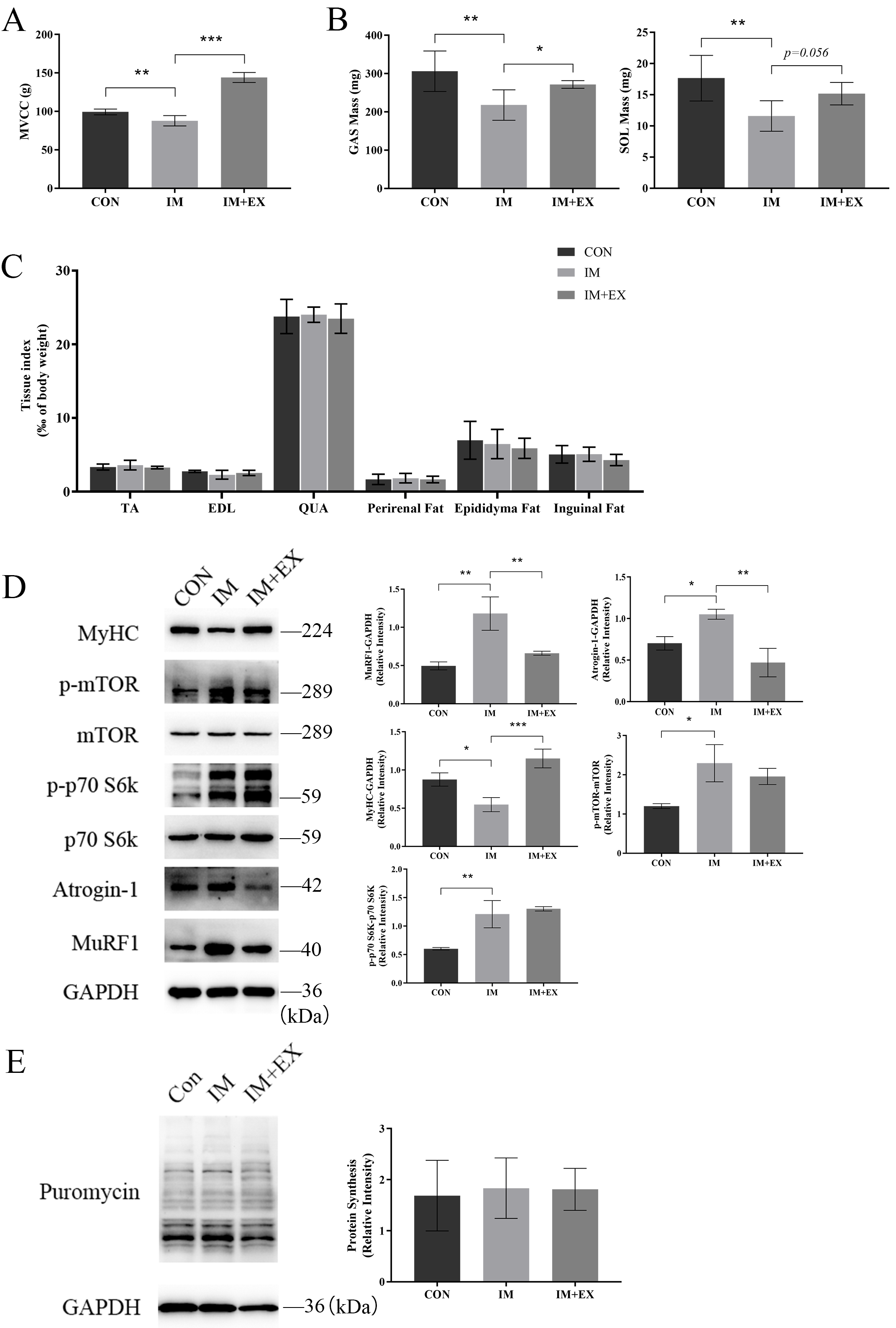

Supplement: Supplementary file 8 — Figure S3: Effects of combined exercise on tissue index and skeletal muscle degradation/synthesis in mice following immobilization. (A) MVCC test. (B) GAS and SOL muscle mass. (C) TA, EDL and QUA muscle and adipose tissue mass adjusted for body weight. (D) Protein degradation and synthesis‐related protein expression were detected by Western blot in GAS muscle. (E) Representative blot and quantification of puromycin incorporation to detect de novo protein synthesis in GAS muscle. One‐way ANOVA was used for A–C (n = 6) and D and E (n = 3). Values are means ± SEM. *p < 0.05; **p < 0.01; ***p < 0.001. TA: tibialis anterior. [file JCSM-16-e70083-s010.tif]

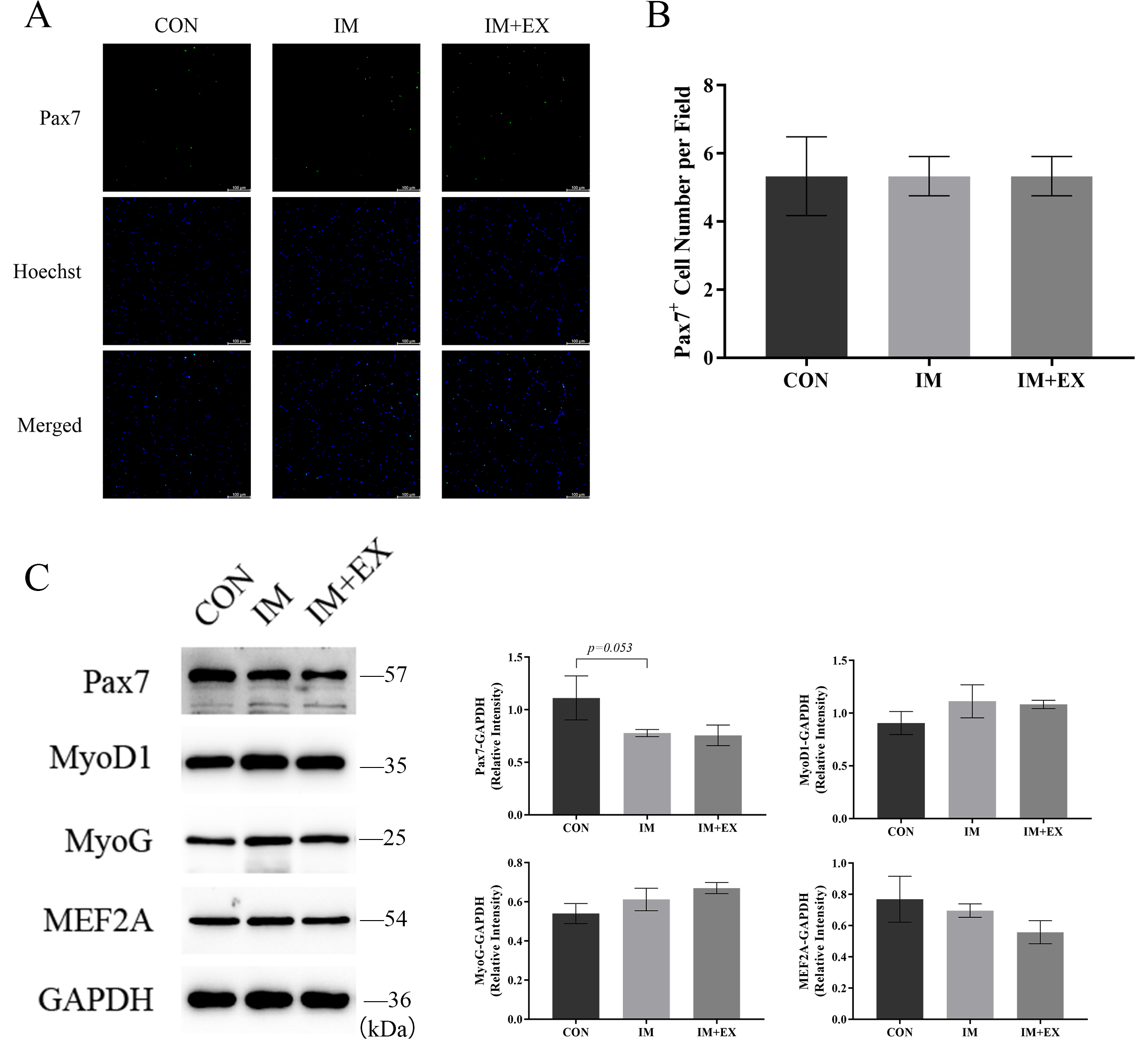

Supplement: Supplementary file 9 — Figure S4: Immobilization and combined exercise did not impair muscle regeneration. (A) Immunofluorescence was used to quantify the number of MuSCs in GAS muscle. Representative images show Pax7 staining (green) and Hoechst nuclear staining (blue). Scale bar: 100 μm. (B) Quantitative analysis of Pax7‐positive cells in GAS muscle. (C) Western blot analysis of proteins related to muscle regeneration in GAS muscle. One‐way ANOVA was used for B and C (n = 3). Values are means ± SEM. MuSCs: muscle stem cells. [file JCSM-16-e70083-s005.tif]

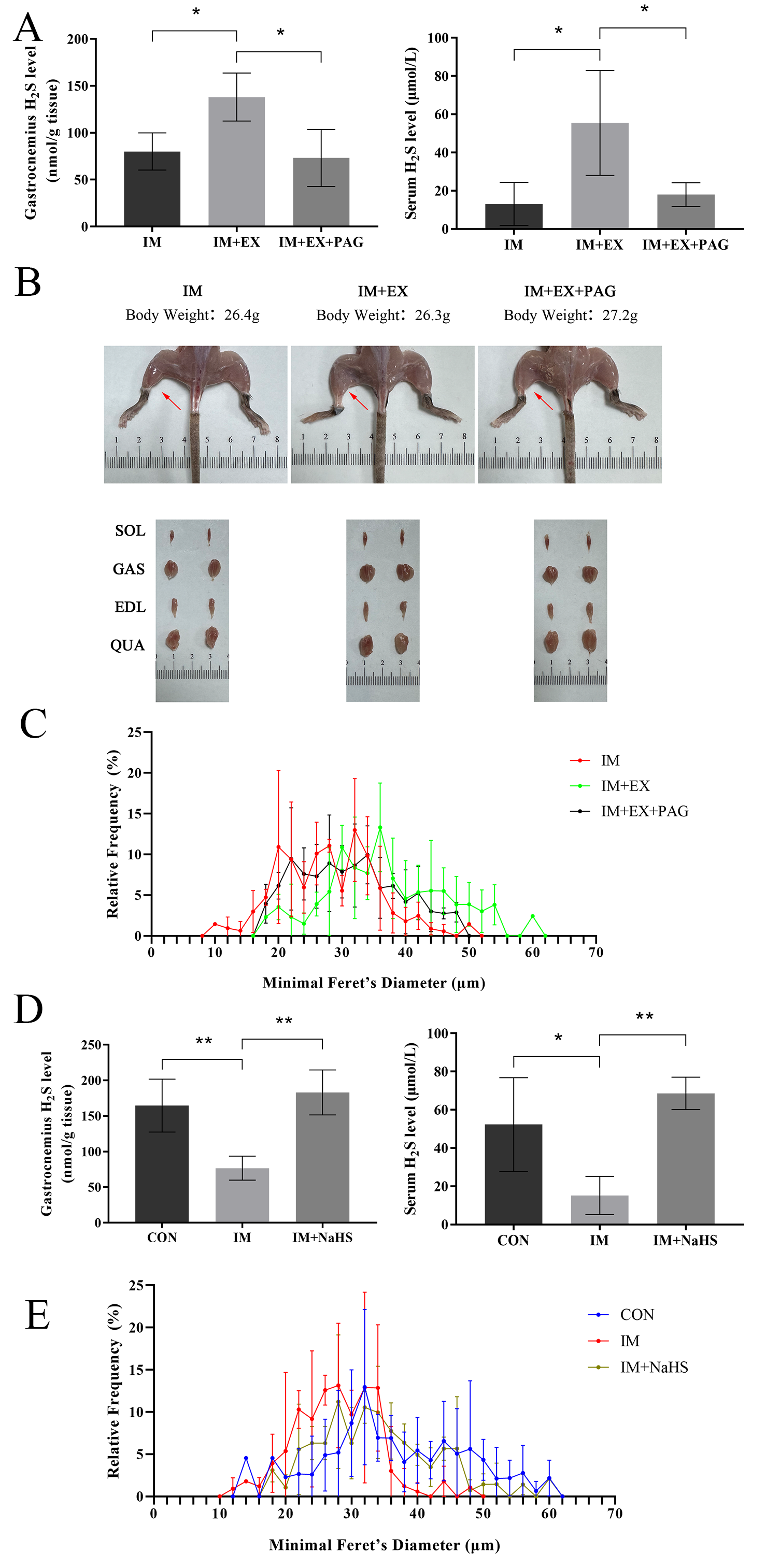

Supplement: Supplementary file 10 — Figure S5: Protective effect of CSE/H2S in disuse muscle atrophy. (A) Effectiveness of CSE‐specific inhibitor PAG. (B) Representative images of hindlimb morphology and muscles in each group after PAG intervention. (C) Distribution of GAS myofiber diameters following PAG intervention. (D) Effectiveness of the H2S donor NaHS. (H) Body weight changes during NaHS intervention. (E) Distribution of GAS myofiber diameters following NaHS intervention. One‐way ANOVA was used for A and D (n = 3–4). Values are means ± SEM. *p < 0.05; **p < 0.01. [file JCSM-16-e70083-s009.tif]

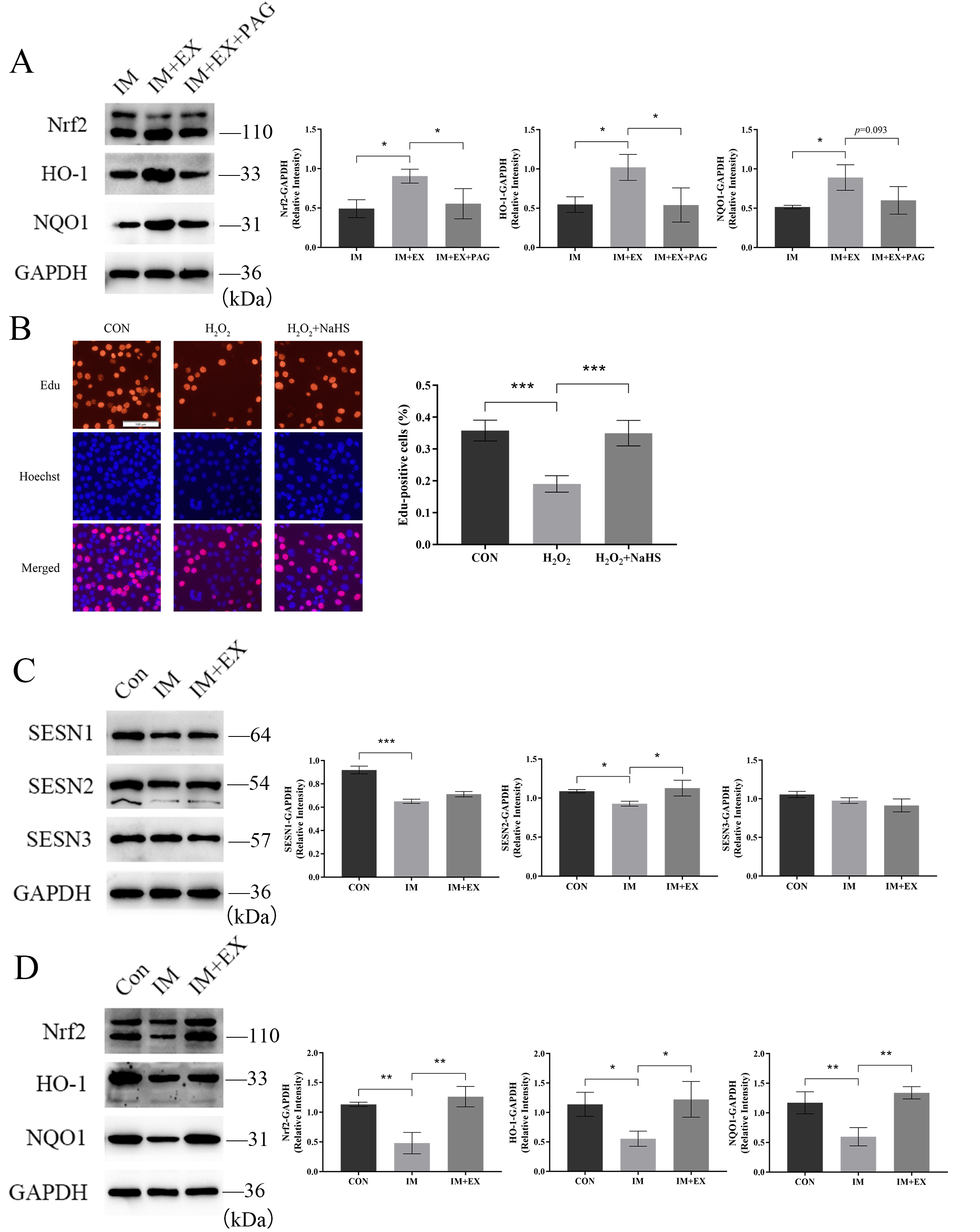

Supplement: Supplementary file 11 — Figure S6: Effects of exercise and PAG intervention on the SESN2‐Nrf2 pathway and restoration of C2C12 myoblasts viability through NaHS treatment. (A) Western blot analysis showed the impact of PAG intervention on oxidative stress‐related protein expression in GAS muscle. (B) Edu staining to assess the proliferative capacity of C2C12 myoblasts, with Edu staining (red) and Hoechst nuclear staining (blue). Scale bar: 100 μm. Quantitative analysis of Edu‐positive cell percentage using Image J. (C) Western blot analysis showing the effect of immobilization and exercise interventions on the expression of the SESN family in GAS muscle. (D) Western blot analysis of the effect of immobilization and exercise intervention on oxidative stress‐related protein expression in GAS muscle. One‐way ANOVA was used for A–D (n = 3–4). Values are means ± SEM. *p < 0.05; **p < 0.01; ***p < 0.001. Edu: 5‐Ethynyl‐20‐deoxyuridine. [file JCSM-16-e70083-s006.tif]
